# Integrative analysis of the lncRNA-miRNA-mRNA interactions in smooth muscle cell phenotypic transitions

**DOI:** 10.3389/fgene.2024.1356558

**Published:** 2024-04-10

**Authors:** Aatish Mahajan, Junyoung Hong, Irene Krukovets, Junchul Shin, Svyatoslav Tkachenko, Cristina Espinosa-Diez, Gary K. Owens, Olga A. Cherepanova

**Affiliations:** ^1^ Department of Cardiovascular and Metabolic Sciences, Lerner Research Institute, Cleveland Clinic, Cleveland, OH, United States; ^2^ Department of Genetics and Genome Sciences, Case Western Reserve University, Cleveland, OH, United States; ^3^ Center for Molecular Medicine and Genetics, Wayne State University, Detroit, MI, United States; ^4^ Robert M. Berne Cardiovascular Research Center, University of Virginia, Charlottesville, VA, United States

**Keywords:** smooth muscle cells, long non-coding RNAs, micro RNAs, integrative analysis, OCT4 activation

## Abstract

**Objectives:** We previously found that the pluripotency factor OCT4 is reactivated in smooth muscle cells (SMC) in human and mouse atherosclerotic plaques and plays an atheroprotective role. Loss of OCT4 in SMC *in vitro* was associated with decreases in SMC migration. However, molecular mechanisms responsible for atheroprotective SMC-OCT4-dependent effects remain unknown.

**Methods:** Since studies in embryonic stem cells demonstrated that OCT4 regulates long non-coding RNAs (lncRNAs) and microRNAs (miRNAs), making them candidates for OCT4 effect mediators, we applied an *in vitro* approach to investigate the interactions between OCT4-regulated lncRNAs, mRNAs, and miRNAs in SMC. We used OCT4 deficient mouse aortic SMC (MASMC) treated with the pro-atherogenic oxidized phospholipid POVPC, which, as we previously demonstrated, suppresses SMC contractile markers and induces SMC migration. Differential expression of lncRNAs, mRNAs, and miRNAs was obtained by lncRNA/mRNA expression array and small-RNA microarray. Long non-coding RNA to mRNA associations were predicted based on their genomic proximity and association with vascular diseases. Given a recently discovered crosstalk between miRNA and lncRNA, we also investigated the association of miRNAs with upregulated/downregulated lncRNA-mRNA pairs.

**Results:** POVPC treatment in SMC resulted in upregulating genes related to the axon guidance and focal adhesion pathways. Knockdown of *Oct4* resulted in differential regulation of pathways associated with phagocytosis. Importantly, these results were consistent with our data showing that OCT4 deficiency attenuated POVPC-induced SMC migration and led to increased phagocytosis. Next, we identified several up- or downregulated lncRNA associated with upregulation of the specific mRNA unique for the OCT4 deficient SMC, including upregulation of ENSMUST00000140952-Hoxb5/6 and ENSMUST00000155531-Zfp652 along with downregulation of ENSMUST00000173605-Parp9 and, ENSMUST00000137236-Zmym1. Finally, we found that many of the downregulated miRNAs were associated with cell migration, including miR-196a-1 and miR-10a, targets of upregulated ENSMUST00000140952, and miR-155 and miR-122, targets of upregulated ENSMUST00000155531. Oppositely, the upregulated miRNAs were anti-migratory and pro-phagocytic, such as miR-10a/b and miR-15a/b, targets of downregulated ENSMUST00000173605, and miR-146a/b and miR-15b targets of ENSMUST00000137236.

**Conclusion:** Our integrative analyses of the lncRNA-miRNA-mRNA interactions in SMC indicated novel potential OCT4-dependent mechanisms that may play a role in SMC phenotypic transitions.

## Graphical Abstract

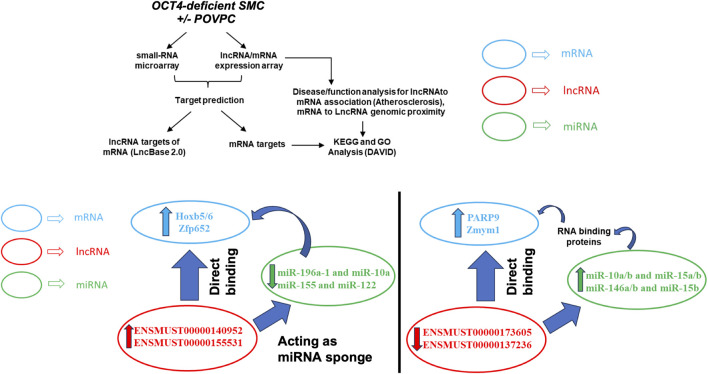

## Introduction

Cardiovascular diseases (CVD) are the leading cause of death, with more than 17.3 million fatalities per year around the globe (McClellan et al., 2019). Atherosclerosis is a major root cause of CVD and accounts for a large proportion of these deaths. This is an inflammatory disease of the arterial wall, resulting in the formation of complex atherosclerotic plaques and leading to multiple thrombotic events, including myocardial infarction and stroke (Libby, 2021). Vascular cells, such as smooth muscle cells (SMC), endothelial cells (EC), and macrophages (MФ), contribute to atherosclerosis plaque development.

There is growing evidence that SMC phenotypic transitions have a critical role in the pathogenesis of cardiovascular diseases. Importantly, recent SMC-lineage tracing and single cell (sc)RNAseq studies revealed extensive plasticity of SMC in atherosclerosis (Dobnikar et al., 2018; Wirka et al., 2019; Alencar et al., 2020; Pan et al., 2020). These include the transition of SMC from differentiated cells expressing high levels of contractile SMC-lineage markers (e.g., MYH11) to several phenotypically modulated states, including inflammatory, МФ-like (Shankman et al., 2015), osteogenic-like (Alencar et al., 2020), or myofibroblast-like phenotypes (Wirka et al., 2019). However, the molecular mechanisms regulating these transitions are not fully understood.

Long non-coding RNAs (lncRNA) have been identified and characterized as a sub-class of non-coding RNAs with lengths longer than 200 nucleotides (nts) playing critical regulatory roles in many human diseases, including atherosclerosis (Li et al., 2016; Chen X. et al., 2017). MicroRNAs (miRNAs) are short RNAs with lengths 21–23 nts that bind to mRNAs and post-transcriptionally regulate gene expression. Another important class of non-coding RNA are circular RNAs (circRNAs), single-stranded and covalently closed RNA molecules, which act as transcriptional regulators and have been found in many different species, including mammals (Zhou et al., 2020). Due to their unique structure, circRNA are more stable than other non-coding RNAs and are found in circulation and urine, making them potential good targets for therapeutics and diagnostics (Zhou et al., 2020). Non-coding RNAs account for more than 98% of the human genome (Zhou et al., 2020) and have significant roles in regulating many cellular functions in normal conditions (e.g., during development or normal tissue homeostasis) and pathological conditions (Xue et al., 2017; Salviano-Silva et al., 2018). A growing number of reports describe the role of miRNA and lncRNA in SMC dysfunction in pathological conditions, including cancer (Poniewierska-Baran et al., 2022; Mazziotta et al., 2023a) and atherosclerosis (Kumar et al., 2019; Zhou et al., 2021). However, these mechanisms have not been fully explored.

Long non-coding RNAs regulate gene expression by multiple mechanisms, including serving as “decoy”, “guide”, or “scaffold” to the critical transcriptional factors of other important regulatory genes in a location-dependent manner (Wilusz et al., 2009). It is also known that lncRNAs are associated with chromatin-modifying complexes and are transcribed from enhancers involved in the phase separation of nuclear condensates and domains. These findings suggest a close relationship between lncRNA expression and the spatial regulation of gene expression during development (Mattick et al., 2023). Most of the lncRNAs are localized in the nuclei, and their nucleus-specific lncRNA functions (i.e., chromosome scaffolding, chromatin remodeling, alternative splicing, epigenetic control of transcription, etc.) have been reported (Derrien et al., 2012; Kugel and Goodrich, 2012; Tripathi et al., 2013). However, lncRNAs are also localized in the cytoplasm, and the functions of these cytoplasmic lncRNAs remain poorly understood. These lncRNAs are transcribed from nuclear DNA or expressed locally in the cytoplasm (e.g., mitochondrial DNA-encoded lncRNAs) (Mercer et al., 2011) and are reported to be involved in maintaining cellular structure and functions (Rashid et al., 2016), mRNA translation and stability, availability of cytoplasmic factors, and scaffolding of proteins (reviewed in *Noh et al.*) (Noh et al., 2018).

One of the regulatory functions of lncRNAs is their ability to act as competitive endogenous RNA (ceRNA) by working as a miRNA “sponge” or as precursor and encoding miRNAs, in turn regulating the effects of miRNA by competing for the miRNA binding to mRNA (McMullen and Drew, 2016). Similar to lncRNAs, circRNAs also exert biological functions by acting as transcriptional regulators, miRNA sponges, and protein templates (Zhou et al., 2020). Due to the complex and complicated nature of interactions between lncRNA, miRNA, and mRNA, multiple groups started to employ integrative analysis between these molecules in different cells and different *in vitro* and *in vivo* conditions (Cabili et al., 2011; Du et al., 2016; Qian et al., 2018; Wang et al., 2019; Zou et al., 2019; He et al., 2022; Liu et al., 2023). In particular relevance to SMC, using integrative analysis of altered lncRNA, miRNA, and mRNA expression, *Chinnappan et al.* (Chinnappan et al., 2019) demonstrated that lncRNA/miRNA/mRNA interactions play a vital role in human pulmonary artery SMC treated with cocaine and HIV-Tat protein. However, similar interactions in the phenotypic transitions of vascular SMC are still unknown.

We recently found that the embryonic stem cell/induced pluripotent stem cell (iPSC) factor OCT4, which was believed to be silenced in somatic cells, plays an atheroprotective role in SMC, in that knockout of *Oct4* in SMC led to marked increases in lesion size and multiple indices of plaque instability in *Apoe* ^−/−^ mice (Cherepanova et al., 2016). It has also been shown that SMC-specific knockout of *Oct4* protects SMC from hyperproliferation after acute vascular injury (Shin et al., 2023). Moreover, we found that knockout of *Oct4* in cultured SMC resulted in decreased cell migration and increased cell proliferation. Mechanistically, we demonstrated that OCT4 directly regulates SMC contractile genes, including ACTA2 and TAGLN. Bulk RNAseq analyses on cultured aortic *Oct4* wild-type and knockout SMC demonstrated significant changes in gene expression profiles (Shin et al., 2023), indicating a broad effect of OCT4 in SMC. However, it is still unknown if lncRNA and/or miRNA contribute to the OCT4-dependent effects in SMC.

Since it has been reported that OCT4 transcriptionally regulates miRNA and lncRNA in embryonic (Shi and Jin, 2010) and cancer cells (Zhang et al., 2020), we hypothesized that similar mechanisms may be activated in SMC. This study aims to identify novel potential OCT4-dependent interactions among lncRNAs, mRNAs, and miRNAs that could be involved in vascular SMC phenotypic changes. Herein, we adopted an integrative analysis approach similar to *Chinnappan et al* (Chinnappan et al., 2019) and others (Cabili et al., 2011; Du et al., 2016; Qian et al., 2018; Wang et al., 2019; Zou et al., 2019; He et al., 2022; Liu et al., 2023) to construct an interactive network of altered lncRNA, miRNA, and mRNA expression in OCT4 deficient mouse aortic SMC (MASMCs) treated with the pro-atherogenic oxidized phospholipid POVPC (1-palmitoyl-2-(5-oxovaleroyl)-*sn*-glycero phosphatidylcholine) that we previously found induces SMC phenotypic switching, including suppression of SMC contractile markers and mediation of SMC proliferation and migration (Pidkovka et al., 2007; Cherepanova et al., 2009). Our studies identified new interactions between OCT4, lncRNA, and miRNA that advance our understanding not only of OCT4-dependent effects but also general causes of SMC phenotypic transitions associated with atherosclerosis.

## Materials and methods

### Cell culture and RNA isolation

Primary mouse aortic smooth muscle cells (MASMC) were isolated and grown as previously described (Cherepanova et al., 2016). Red blood cells (RBC) were isolated from the blood of the young (10 weeks of age) C57B6 mice as previously described (Hanson et al., 2008) and immediately used for phagocytosis experiments. The animal study were approved by the University of Virginia (protocol 2500) and Lerner Research Institute Animal Care and Use Committees (protocol 2433). The study was conducted in accordance with the local legislation and institutional requirements. For experiments, cells at the 70% confluency were transfected with blocking si*Oct4* or non-target siRNA (siNT) (Dharmacon) for 48 h in serum-free transfection media (Dharmacon), followed by the treatment with 10 μg/mL POVPC (Cayman Chem, #10031) or vehicle (DMSO) for 24 h based on our previous findings (Pidkovka et al., 2007). At the end of the experiment, cells were harvested with the Trizol reagent (Invitrogen), which preserves most of the small RNAs, including lncRNAs and miRNAs, and the total RNA was isolated according to the manufacturer’s instructions.

### Quantitative real time (qRT)-PCR

The cDNA was prepared using an iScript gDNA Clear cDNA Synthesis Kit (BioRad). Quantitative RT-PCR was performed using RT2 SYBR Green ROX qPCR Master Mix kit (Cat#330522, Qiagen) for *Oct4* exon 1 primers or SensiFAST™ SYBR NO-ROX Mix (Bioline) for *18s RNA* and lncRNAs. The qRT-PCR primers for *Oct4* exon1 and *18s RNA* were previously described (Cherepanova et al., 2016). For lncRNAs, we used primers as ENSMUST00000140952 (F: CACCCCAGCCCGGTAAAC R: CAT​GGG​CGA​TCC​ACA​TGA​A) and ENSMUST00000173605 (F: TTC​CTT​CTG​CGT​CAG​TAT​CAT​CTT R: CCA​AGC​TGC​TTT​GCT​CAA​ATT).

### Mouse lncRNA/mRNA and small RNA expression arrays

Long non-coding RNA/mRNA and small RNA microarrays were performed by Arraystar Inc. on the same total RNA samples isolated from MASMC transfected with either siRNA to *Oct4* or NT pooled siRNA (Dharmacon) and treated with either DMSO or POVPC (10 μg/mL) for 24 h.

#### Long non-coding RNA/mRNA

After sample quality controls using Nanodrop ND 1000 and determining RNA integrity using denaturing gel electrophoresis, each sample was amplified and transcribed into fluorescent cRNA along the entire length of the transcripts without 3′bias utilizing a random priming method (Array Star Flash RNA Labeling Kit, Arraystar Inc.). The labeled cRNAs were hybridized onto the mouse LncRNA Array v4.0 (8 × 60 K, Arraystar). These microarrays are highly sensitive and accurate even for low abundance lncRNAs (the quantitative efficiency is as low as 1 transcript per cell). These oligo-based probes hybridize the target RNA at high affinity, independent of other abundant RNAs. Also, the design of the transcript-specific array probe is based on well-established transcript models for every lncRNA isoform, ensuring clear and precise isoform detection and quantification.

#### Small RNA array

For each sample, 100 ng total RNA was firstly dephosphorylated to form the 3-OH end. The 3-OH-ended RNA was then denatured by DMSO and enzymatically labeled with Cy3. This ligation of small RNAs with Cy3 dye directly at their 3′-OH ends, completely avoids RNA pretreatments to remove internal RNA modifications, abortive reverse transcription due to the modifications and RNA fold hindrance, and skewed PCR amplification steps (Yuan et al., 2022). All these help to preserve the fidelity of native small RNA levels and achieve the unbiased high quantification accuracy. The labeled RNA was hybridized onto Arraystar Mouse small RNA Microarray (8 × 15K, Arraystar).

The Arrays were scanned by the Agilent Scanner G2505C. Agilent Feature Extraction software (version 11.0.1.1) was used to analyze the acquired array images. Quantile normalization and subsequent data processing were performed using the GeneSpring GX v12.1 software package (Agilent Technologies). Arraystar Small RNA Array uses high-affinity probe hybridization to achieve very high sensitivity even for small RNAs at low abundance. The smart probe design incorporates a 5′-hairpin structure and normalized sequence targeting region to specifically distinguish small RNAs with only 1–2 nucleotide differences.

### Differential expression analyses

#### Differential expression analyses were performed after quality control analyses

##### Long non-coding RNA/mRNA

Differentially expressed lncRNAs and mRNAs with statistical significance were identified through Fold Change filtering between two samples. Differentially expressed LncRNAs and mRNAs with statistical significance between siNT DMSO vs. siNT POVPC (*effect of POVPC in wild type SMC*), siOCT4-DMSO vs. siNT-DMSO (*effect of OCT4 inactivation*), and siOCT4 POVPC vs. siOCT4 DMSO (*effect of POVPC in OCT4 deficient SMC*) groups were identified through Volcano filtering between two groups. Finally, hierarchical clustering was performed to show the distinguishable lncRNAs’ and mRNAs’ expression patterns among samples. The Ingenuity Pathway Analysis (IPA; QIAGEN Inc.) was used to analyze the associated pathways. The statistical significance of a gene association with biological function or pathway was determined using the right-tailed Fisher’s Exact Test.

##### Small RNAs

Differentially expressed small RNAs between two comparison groups were identified by fold change (FC) of ≥1.5 and statistical significance (*p*-value) of ≤0.05 thresholds. Hierarchical clustering heatmaps, scatter plots, and volcano plots were plotted to display small RNAs expression patterns among samples by R software. Pathway analysis (KEGG; Kyoto Encyclopedia of Genes and Genomes) and GO analysis (Gene Ontology) (Mazziotta et al., 2023b) were applied to determine these differentially expressed mRNAs’ roles in the biological pathways or GO terms.

#### Integrative analysis of dysregulated non-coding RNAs and mRNAs

The integrative analysis was adopted from *Chinnappan et al.* (reference 29) and modified for our experimental design. For analysis, mRNA, miRNA, and lncRNA that were differentially expressed with >1.5-fold up- or downregulated between treatment groups (NT DMSO, NT POVPC, siOCT4 DMSO, siOCT4 POVPC) and *p*-values less than 0.05 were used. The IPA software was used to investigate the relationship between differentially expressed miRNAs and mRNAs. First—The initial analysis was performed by only taking experimentally validated interactions of miRNA/mRNA, and later, the mRNA expression data were included to take into account the interactions of highly predictive and experimentally validated miRNA/mRNA associations. For the experimentally validated miRNA/mRNA interactions, we utilized the integrated information from TarBase and miRecords (IPA), and for predicted miRNA/mRNA interactions, we used information from TargetScan (IPA). Second—The regulatory relationships between differentially expressed miRNA and lncRNA were extracted from LncBase v2.0. using DIANA Tools (Paraskevopoulou et al., 2016). Only interactions that were experimentally validated were taken into account. Third—Pathway enrichment analysis was performed using IPA software and DAVID (Database for Annotation, Visualization, and Integrated Discovery) software (Huang da et al., 2009). Long non-coding RNAs, which were differentially expressed, were annotated for diseases and biological functions using the LncRNADisease database (Wang et al., 2016).

#### LncRNA conservation analysis

To assess the potential conservation of selected lncRNAs across species, phyloP analysis (PHAST (cshl.edu) was employed to quantify evolutionary conservation by comparing the sequence of interest to placental mammals and vertebrates, in conjunction with the multiz alignment function from the UCSC genome browser (Kent et al., 2002). Blastn (Basic Local Alignment Search Tool for nucleotides) analysis was conducted to determine human orthologs by querying the lncRNA sequences against the *Homo Sapiens* genomic and transcript database. Subsequently, the identified lncRNA sequences and their putative orthologs were subjected to alignment using SnapGene software for validation.

#### SMC migration assay

For experiments, cells at the 70% confluency were transfected with blocking siOct4 or non-target siRNA (NT siRNA) for 48 h in serum-free media, followed by the Boyden chamber transmigration assay in response to different concentrations of POVCP, as previously described (Cherepanova et al., 2016).

#### Phagocytosis assay

Phagocytosis assay was performed as previously described in *Kolb et al.* (Kolb et al., 2007). Briefly, *Oct4* wild type and knockout SMC(34) were plated in 24 well plates. Cells at 70% confluency were growth arrested with serum-free media with supplements (DMEM/F12 [Gibco], 100 U/mL penicillin/streptomycin [Gibco], 1.6 mM/L L-glutamine [Gibco], L-ascorbic acid 0.2 mM [Sigma], transferrin 5 μg/mL [Sigma], insulin 2.8 μg/mL [Sigma], Na-selenate 6.25 ng/mL [Sigma]) for 24 h and then treated with 0 or 40 μg/mL of oxidized low-density lipoprotein particles (oxLDL) (Biomedical Thechnologies) for 3 days. At day 4, mouse RBSs were plated (∼1 × 10^5^ cells/mL) on the top of SMC for 24 h. Media was aspirated, and cells were washed with PBS. The membrane-bound RBCs were lysed by hypotonic shock (1 min in distilled sterile H_2_O on ice). SMCs containing phagocytosed RBCs were lysed by 1% Triton-X100 in PBS. Hemoglobin was then separately analyzed in hypotonic (H_2_O)- and Triton-lysate fractions using 2-7-diaminofluorene reagent (Sigma).

### Statistical analysis

For the functional *in vitro* experiments, the normality of the data was checked using Kolmogorov-Smirnov test. One-way analysis of variance (ANOVA) was used to compare two groups of continuous variables with normal distribution, and two-way ANOVA, followed by *post hoc* tests, was used to compare multiple group comparisons as previously described (Shin et al., 2023). *p* < 0.05 was considered significant. GraphPad software 9.4.0 was used for all statistical analyses.

## Results

### Differential expression of lncRNA in OCT4-deficient MASMC

**FIGURE 1:**
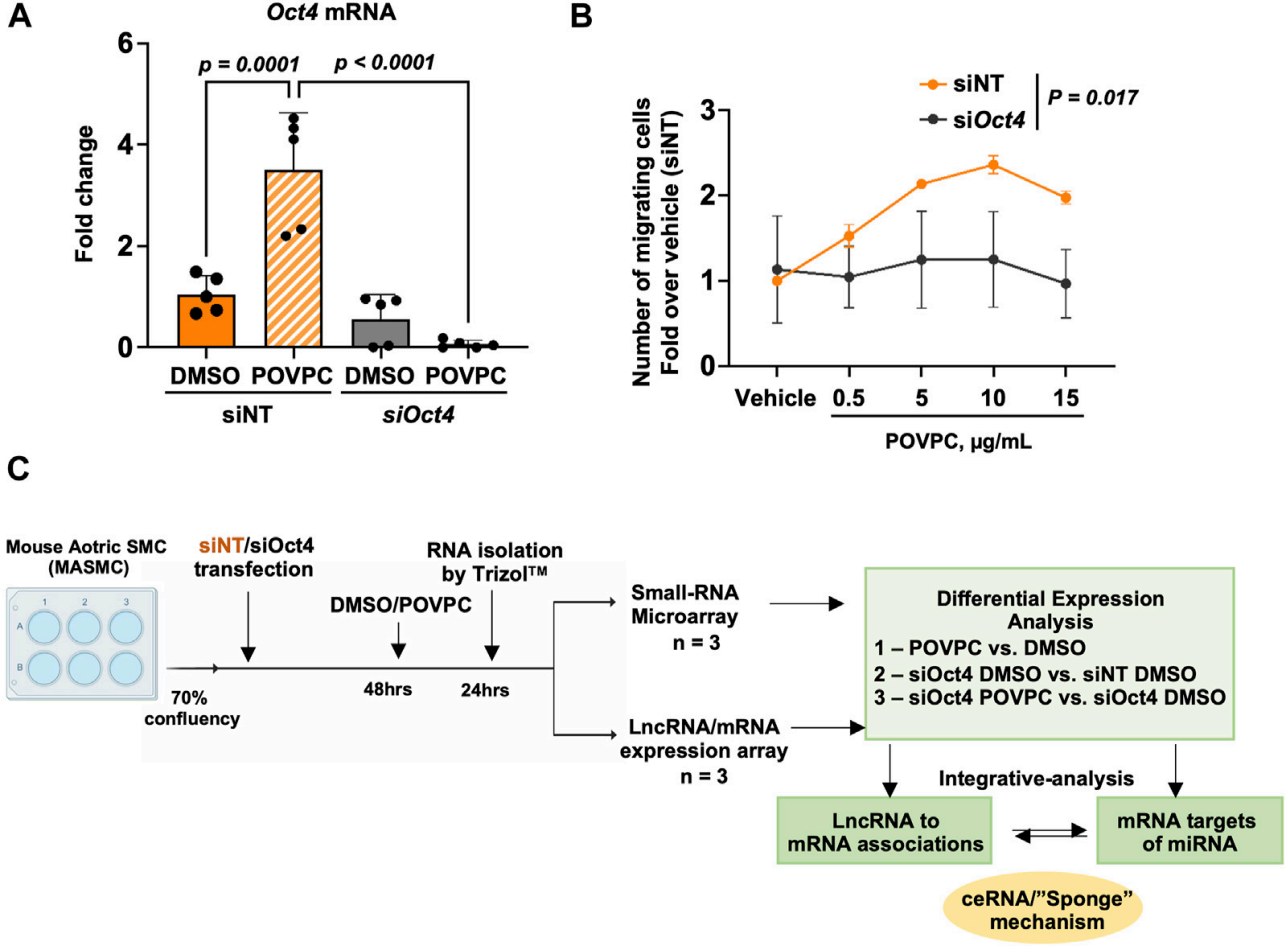
Experimental design. **(A)** Quantitative RT-PCR analysis to validate the knockdown of *Oct4* in mouse aortic SMC (MASMC) transfected with blocking si*Oct4* or non-target siRNA (siNT), followed by the treatment with the pro-atherogenic oxidized phospholipid POVPC. Values = means ± s. e.m. *p* values were quantified by two-way ANOVA, followed by Tukey *post hoc* test; *n* = 5 biological replicates from 3 independent experiments. **(B)** Oct4 knockdown (*siOct4*) attenuated POVPC-induced SMC migration. *p* values for siNT vs. si*Oct4* across all concentrations of POVPC were quantified by two-way ANOVA; *n* = 3 independent experiments. **(C)** The schematic shows the overall workflow of the analysis. DAVID—The Database for Annotation, Visualization, and Integrated Discovery (DAVID) v6.8.

To investigate the effects of OCT4, we chose to use an siRNA knockdown cell culture model to avoid any long-term effects of OCT4 knockout during cell culture. Mouse aortic SMC were transfected with blocking si*Oct4* or control non-target siRNA (siNT) followed by treatment with the oxidized phospholipid POVPC. The knockdown efficiency was confirmed by using qRT-PCR (Figure 1A). We recently demonstrated that transient siRNA knockdown and genetic knockout have similar effects on SMC proliferation and expression of SMC contractile genes (Shin et al., 2023). We also found that similarly to the genetic knockout, inhibiting *Oct4* by blocking siRNA resulted in decreases in SMC trans-migration in response to POVPC (Figure 1B), indicating that siOct4 knockdown is a good cell culture model to study immediate OCT4-dependent mechanisms in SMC.

**FIGURE 2:**
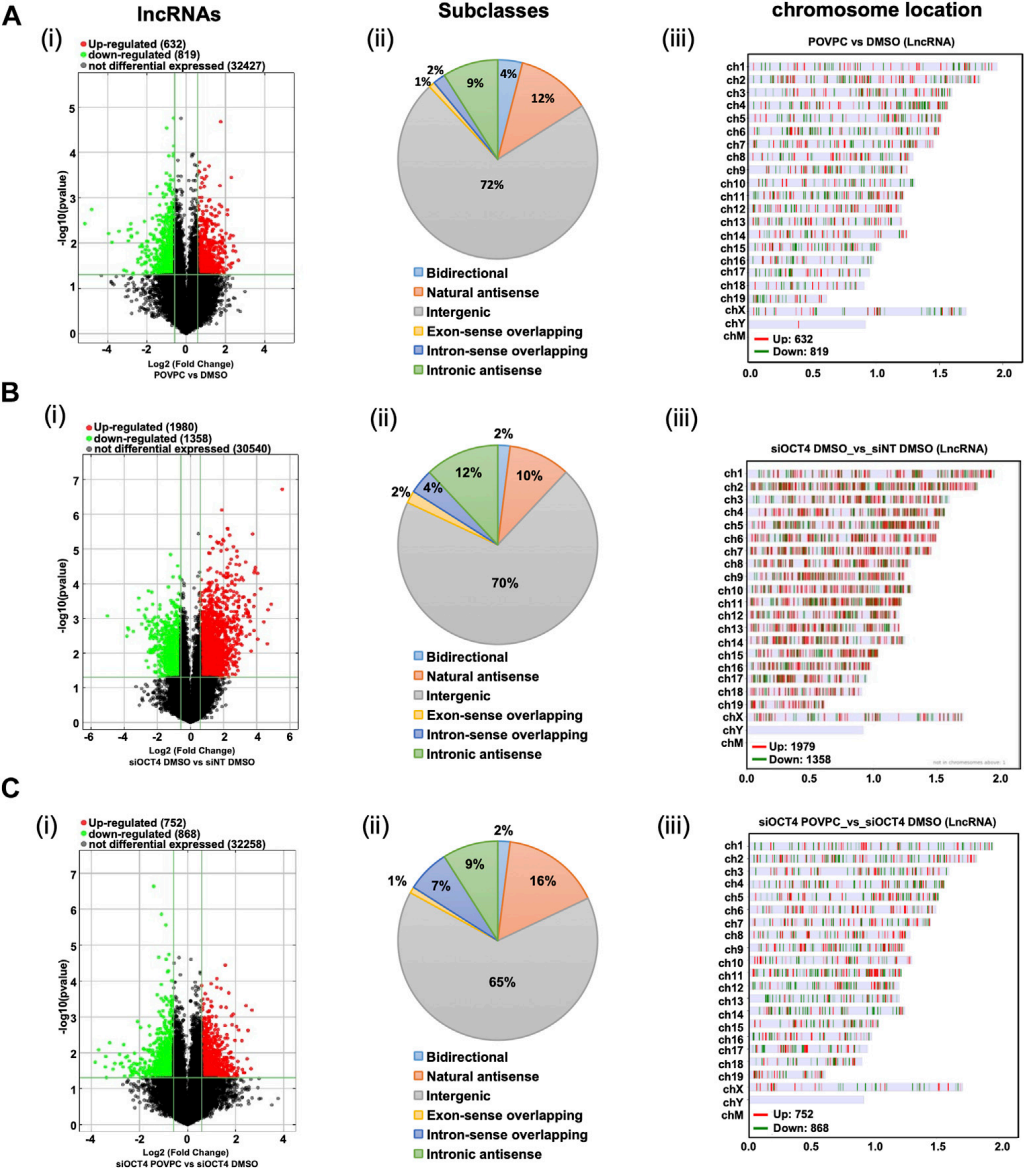
Differential expression and characteristics of lncRNAs**. (A)** POVPC vs. DMSO treatment groups (effect of POVPC in wild type SMC), **(B)** siOCT4-DMSO vs. siNT-DMSO groups (effect of OCT4 inactivation), **(C)** siOCT4-POVPC vs. siOCT4-DMSO groups (effect of POVPC in OCT4 deficient SMC). Volcano plot for significantly dysregulated lncRNAs (i), chromosome location (ii), and Subclasses (iii).

Next, as shown in the schematic diagram in Figure 1C, expression arrays were used to capture lncRNA/mRNA and small-RNA expression profiles in treated MASMC. Analyses identified multiple differentially expressed lncRNAs (Figure 2; Supplementary Table S1). A total of 632 lncRNA were significantly upregulated, and a total of 819 were downregulated in POVPC vs. DMSO treatment groups (*effect of POVPC in wild type SMC*) (Figure 2Ai), 1980 lncRNA were significantly upregulated, and 1,358 were downregulated in siOCT4-DMSO vs. siNT-DMSO groups (*effect of OCT4 inactivation*) (Figure 2Bi), and 752 lncRNAs were significantly upregulated, and 868 were downregulated in siOCT4-POVPC vs. siOCT4-DMSO groups (*effect of POVPC in OCT4 deficient SMC*) (Figure 2Ci). According to their location in genomic sequences and association with the neighboring gene regions, lncRNAs have been categorized into various classes to identify potential mechanisms of action for these molecules in regulating other genes (Herrera-Solorio et al., 2017), (Figure 2A–Cii). Using this categorization, differentially expressed lncRNAs were found to be 72% intergenic, 12% natural antisense, and 9% intronic antisense regions in POVPC vs. DMSO treatment groups, 70% intergenic, 10% natural antisense, and 12% intronic antisense regions in siOCT4-DMSO vs. siNT-DMSO groups, and 65% intergenic, 16% natural antisense, and 9% intronic antisense regions in siOCT4-POVPC vs. siOCT4-DMSO groups (Figure 2A–Cii) in the genome. Also, the analysis revealed that these lncRNAs were widely dispersed over all chromosomes (Figure 2A–Ciii).

These data indicate that transient knockdown of *Oct4* in SMC results in robust dysregulation of lncRNAs, and these OCT4-dependent changes in lncRNA expression are higher than changes in lncRNA expression in response to the oxidized phospholipid POVPC treatment.

#### Differential expression of mRNA in MASMC

**FIGURE 3:**
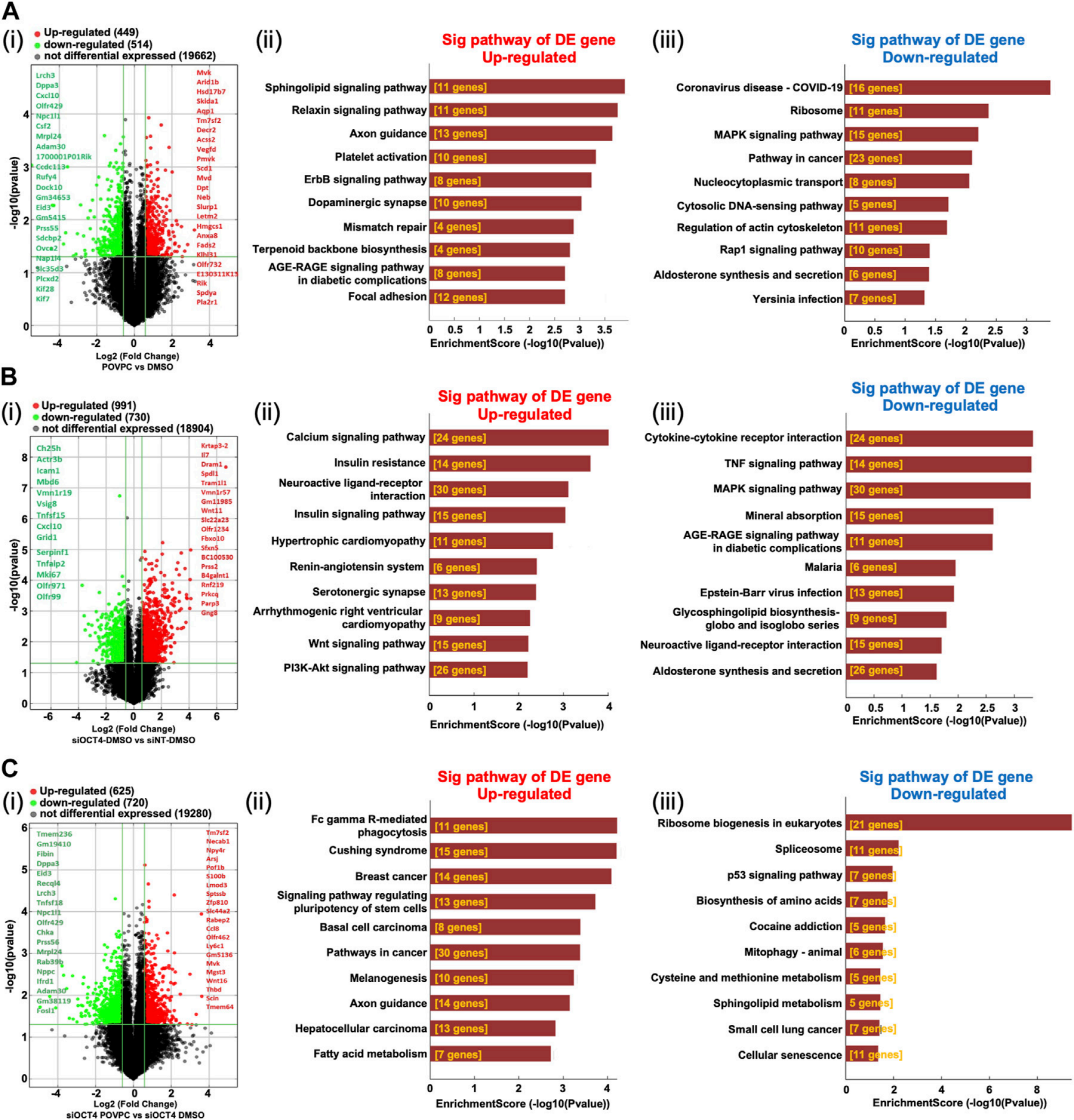
Differential expression and pathway analysis of mRNAs **(A)** POVPC vs. DMSO treatment groups (*effect of POVPC in wild type SMC*), **(B)** siOCT4-DMSO vs. siNT-DMSO groups (*effect of OCT4 inactivation*), **(C)** siOCT4-POVPC vs. siOCT4-DMSO groups (*effect of POVPC in OCT4 deficient SMC*). Volcano plot (i) and KEGG analysis for upregulated (ii) and downregulated (iii) mRNAs.

Analysis of the mRNA expression levels (Supplementary Table S2) in the POVPC vs. DMSO treatment groups showed that 449 mRNAs were upregulated and 514 mRNAs were downregulated (Figure 3Ai), 991 up and 730 down in the siOCT4-DMSO vs. siNT-DMSO groups (Figure 3Bi), and 625 up and 720 down in the siOCT4-POVPC vs. siOCT4-DMSO groups (Figure 3Ci). To estimate the potential of the significantly differently expressed mRNAs to contribute to the phenotypic alterations in MASMC following OCT4 knockdown and POVPC administration, biological functional and pathway analysis was performed based on the GO terms and KEGG pathways.

For POVPC vs. DMSO groups, the *axon guidance* and *focal adhesion* pathways appeared among the top upregulated KEGG pathways (Figure 3Aii). Moreover, the *regulation of cell adhesion* (GO:0030155) and *cell migration* (GO:0030335) GO terms were linked to both up- and downregulated genes. The *COVID-19* and *cancer pathways* were significantly enriched among the downregulated genes in the POVPC-treated cells compared to the control group (Figure 3iii). For the siOCT4-DMSO vs. siNT-DMSO groups, the *insulin* and *calcium signaling* pathways appeared among the top upregulated KEGG pathways (Figure 3Bii), whereas the *TNF signaling* and *cytokine-cytokine receptor* pathways were enriched among the top downregulated pathways (Figure 3BIii). For siOCT4-POVPC vs. siOCT4-DMSO groups, the *cancer* pathways and *phagocytosis* KEGG pathways were upregulated (Figure 3Cii), whereas the *ribosome biogenesis in eukaryotes*, *spliceosome*, *and cellular senescence* pathways were downregulated (Figure 3Ciii). The *ribosome biogenesis* (GO:0042254) and *mesenchymal cell differentiation* (GO:0030154) GO terms were linked to both up- and downregulated genes.

**FIGURE 4:**
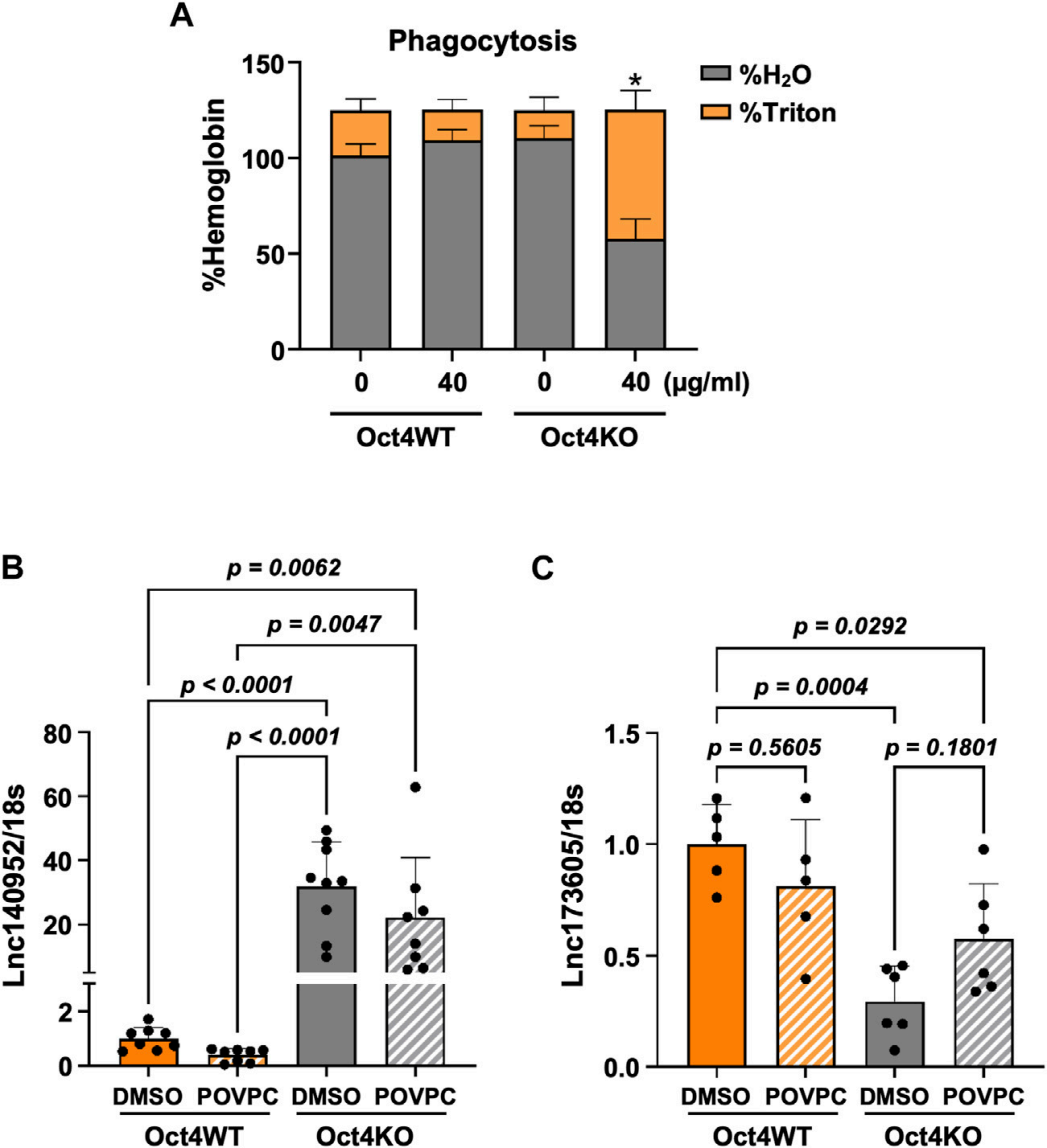
Loss of OCT4 in MASMC resulted in increased phagocytosis and differential expression of lncRNAs ENSMUST00000140952 (*Hoxb5os*) and ENSMUST00000173605. **(A)** *Oct4* wild-type (WT) and knockout (KO) MASMC were treated with oxLDL for 3 days. At day four, mouse red blood cells (RBSs) were plated on the top of SMC for 24 h. The membrane-bound RBCs were lysed by hypotonic shock (1 min in water on ice). MASMCs containing phagocytosed RBCs were lysed by 1% Triton-X100 in PBS. Hemoglobin was then separately assayed in hypotonic (H_2_O) and Triton lysates using 2-7-diaminofluorene reagent. *p < 0.05 by two-way ANOVA, followed by Tukey *post hoc* test (for differences in hemoglobin in the Triton fractions); *n* = 3 independent experiments. **(B,C)** Quantitative RT-PCR showing relative levels of ENSMUST00000140952 **(B)** and ENSMUST00000173605 **(C)** in *Oct4* WT and KO MASMSc treated with POVPC. *p* values were quantified by two-way ANOVA, followed by Tukey *post hoc* test; *n* = 5 biological replicates from 3 independent experiments.

Importantly, these results were consistent with our data showing that OCT4 deficiency attenuated POVPC-induced SMC migration (Figure 1B) and led to increased phagocytosis (Figure 4A).

#### Analysis of the functional relationships between lncRNAs and mRNAs in Oct4-deficient MASMCs

**FIGURE 5:**
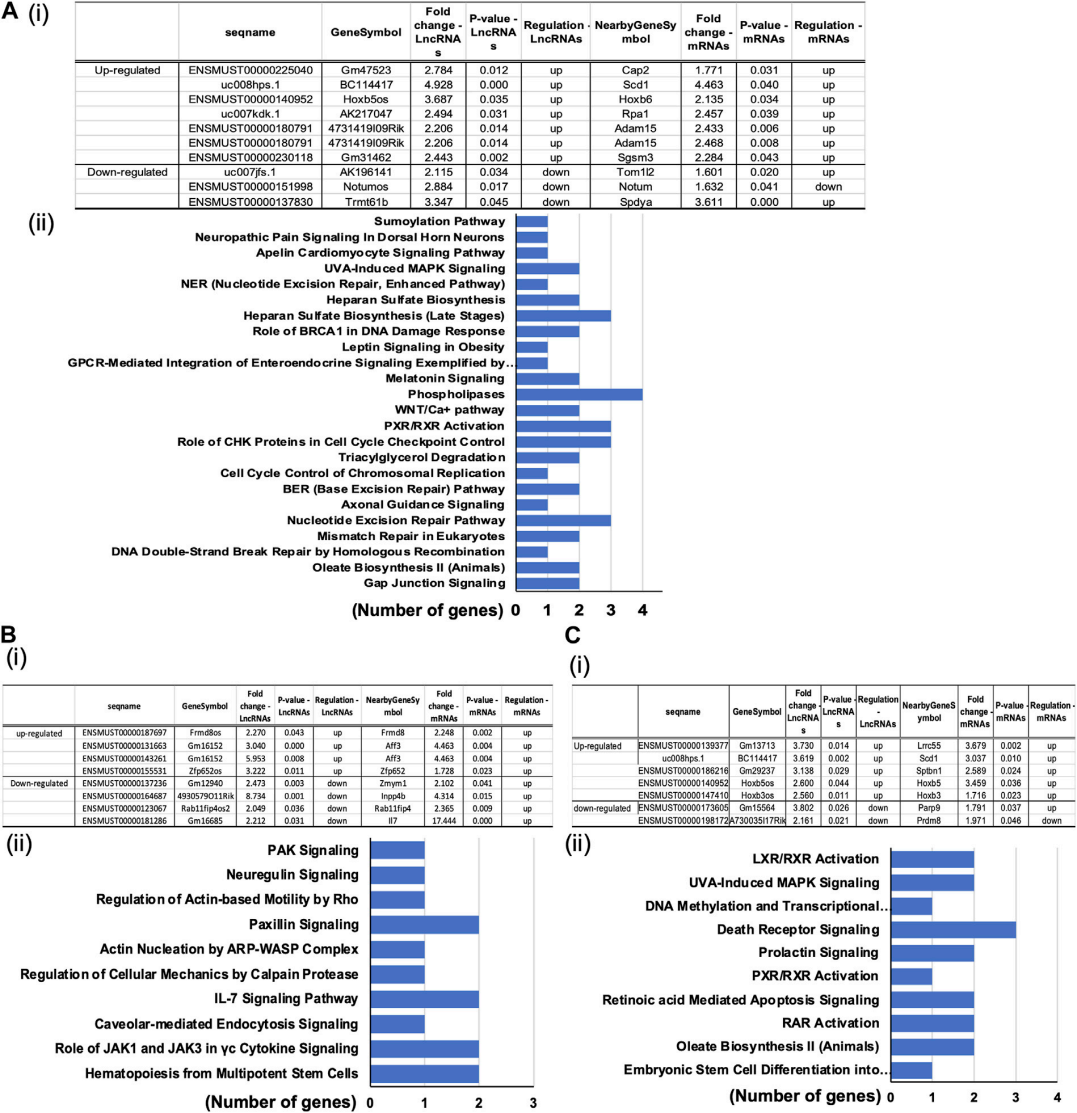
Predicted association of dysregulated lncRNAs to altered mRNA expression**. (A)** POVPC vs. DMSO treatment groups (effect of POVPC in wild type SMC), **(B)** siOCT4-DMSO vs. siNT-DMSO groups (effect of OCT4 inactivation), **(C)** siOCT4-POVPC vs. siOCT4-DMSO groups (effect of POVPC in OCT4 deficient SMC). Table lists microarray details of selected top lncRNA/mRNA predicted associations along with (i); KEGG pathway enrichment for mRNAs associated with lncRNAs based on the genomic proximity (<300 kbp) from IPA analysis (ii).

To determine the reported associations between the significantly differentially expressed mRNAs and lncRNAs and their established functional importance, we searched the LncDisease database. We examined the relationships between all mRNAs and lncRNAs that significantly altered in the treated group compared to the controls. Our analysis revealed 37 highly dysregulated lncRNAs connected to several differently expressed CVD-related mRNAs. The top candidates in the POVPC vs. DMSO groups were the upregulated lncRNA ENSMUST00000140952 linked to the elevated *Hoxb6* mRNA and downregulated lncRNA ENSMUST00000151998 associated with downregulation of the nearby gene *Notum* (Figure 5Ai). In siOCT4-DMSO vs. si-NT-DMSO groups, the upregulated lncRNA ENSMUST00000131663 was associated with upregulated *Aff3*, upregulated ENSMUST00000155531 was associated with upregulated *Zfp652*, whereas downregulated ENSMUST00000137236 was associated with upregulation of *Zmym1* (Figure 5Bi). In siOCT4-POVPC vs. siOCT4-DMSO groups (Figure 5Ci), upregulated ENSMUST00000140952 was associated with upregulated *Hoxb5,* whereas downregulated ENSMUST00000173605 was associated with upregulated *Parp9*.

Furthermore, we analyzed the evolutionary conservation of these lncRNAs to assess whether these associations and potential mechanisms are conserved among vertebrates. The lncRNAs ENSMUST00000140952, ENSMUST00000155531, and ENSMUST00000137236 exhibit significant conservation across mammals, as calculated by phyloP (PHAST, cshl.edu), and UCSC browser multiz alignment, indicating the presence of potential orthologs with similar functions in other species (Kent et al., 2002). In contrast, ENSMUST00000173605 did not demonstrate any conservation (Supplementary Figure S1). Blast alignment of the three conserved lncRNAs identified three potential human orthologs: HOXB3-AS3, ZNF652-AS1, and ZMYM1, respectively. These orthologs not only display partial sequence conservation but are also positionally conserved within their genomic regions (Supplementary Figures S2–S4). Notably, the ZMYM1 gene is a protein-coding gene, but it has six non-protein processed transcripts that partially align (47%) with ENSMUST00000137236, suggesting they could function as antisense regulators for ZMYM1. While the conservation of ENSMUST00000140952.2 associated gene *Hoxb5os* with the human gene HOXB3-AS3 has been previously described (Papaioannou et al., 2019; Degani et al., 2021), further experimental validation of these bioinformatic predictions will be necessary to confirm the roles of ZNF652-AS1 and ZMYM1.

To validate our results further, we used our previously published bulk RNA sequencing data from OCT4 wild-type and knockout MASMC treated with POVPC (GSE75044) (Cherepanova et al., 2016). Importantly, despite the different OCT4 deficiency conditions (permanent long-term *Oct4* knockout vs. transient siRNA-induced knockdown), we found that genes that were identified as targets of the lncRNA in our current study, were also significantly upregulated in the OCT4 knockout cells, including upregulation of *Hoxb5/6*, *Aff3*, and *Zfp652.* In contrast*, Notum* was upregulated in wild-type cells in response to POVPC treatment (Supplementary Figure S5). Moreover, quantitative RT-PCR showed marked increases in ENSMUST00000140952 (Figure 4B) and decreases in ENSMUST00000173605 (Figure 4C) in *Oct4* knockout cells compared to wild-type SMC.

We examined the lncRNA and mRNA array data to find lncRNAs that may act as *cis* (promoter-associated) regulators of mRNAs since lncRNAs are known to regulate the expression of proximal protein-coding genes through *cis*-regulatory mechanisms (Marchese et al., 2017). Based on their genetic proximity (less than 300 kbp), 135 pairs of lncRNA-mRNA interactions were identified for the statistically differentially expressed lncRNAs, including antisense lncRNAs in all three experimental groups (Supplementary Table S3). Interestingly, the highest number of lncRNA-mRNA pairs was observed in the siOCT4-DMSO vs. siNT-DMSO groups (89 pairs vs. 23 pairs in other studied groups), indicating that the effect of OCT4 is a primary driver of the observed lncRNA/mRNA interactions.

Functional analysis of these mRNAs by the DAVID tool showed their connection to several functional pathways, including Phospholipases, Cell cycle control, and Nucleotide excision repair pathways (Figure 5Aii) in POVPC vs. DMSO groups. Pathways related to the Paxillin signaling, IL-7 signaling, and The role of JAK1 and JAK3 in cytokine signaling were some of the enriched pathways in the siOCT4-DMSO vs. siNT-DMSO groups (Figure 5Bii). Further, in siOCT4-POVPC vs. siOCT4-DMSO groups, enrichment of pathways related to the *LXR/RAR* receptor signaling and Apoptosis were observed Figure 5Cii).

#### Dysregulation of miRNAs in Oct4 deficient MASMCs

**FIGURE 6:**
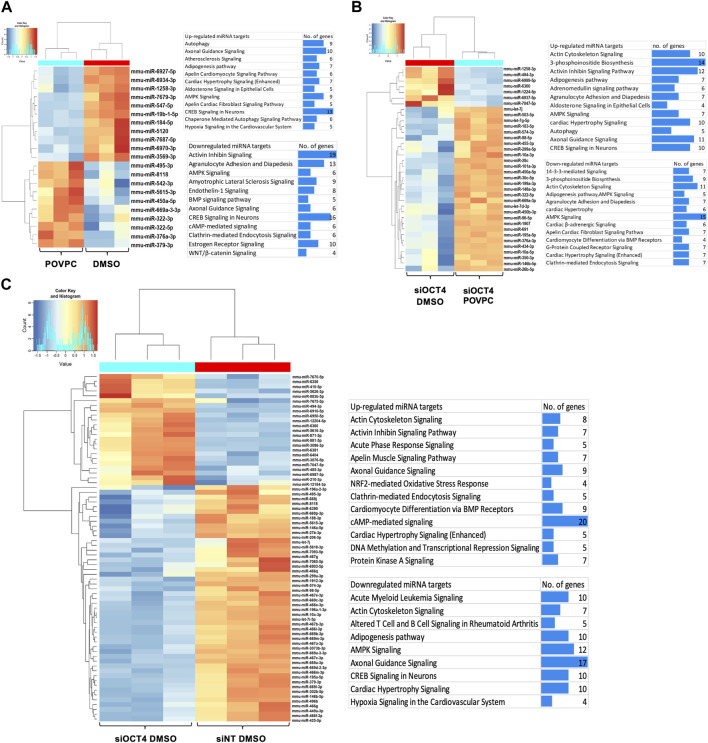
Differential expression of miRNAs and their predicted and experimentally validated mRNA targets in MASMCs **(A)** POVPC vs. DMSO treatment groups (effect of POVPC in wild type SMC), **(B)** siOCT4-DMSO vs. siNT-DMSO groups (effect of OCT4 inactivation), **(C)** siOCT4-POVPC vs. siOCT4-DMSO groups (effect of POVPC in OCT4 deficient SMC). Hierarchical clustering for differentially expressed miRNAs (i); biological functional analysis of significantly dysregulated miRNAs using IPA tool on all the known downstream mRNA targets of these miRNAs (i).

A previous study performed a co-analysis of miRNA/mRNA in SMC dedifferentiation and found that a third of transcripts are regulated by miRNA (Du et al., 2022). To identify OCT4-dependent miRNAs, we performed a small RNA microarray on RNA isolated from MASMC transfected with a non-targeted pool and siRNA-OCT4 and treated with DMSO/POVPC for 24 h (Supplementary Table S4). Differential expression analysis identified 21 miRNAs in the POVPC vs. DMSO groups (Figure 6Ai, ii), 72 miRNAs in the siOCT4-DMSO vs. siNT-DMSO (Figure 6Bi, ii) groups, and 36 miRNAs in siOCT4-POVPC vs. siOCT4-DMSO (Figure 6Ci, ii. Supplementary Figure S6 shows the volcano plots for all the significantly dysregulated miRNAs.

Differentially expressed miRNAs and mRNAs in different experimental groups were subjected to miRNA/mRNA cross-analyses. First, we identified these miRNAs’ known downstream target genes using the IPA miRNA target filtering tool. Then, we used DAVID to perform a biological functional analysis on these genes to determine which genes are regulated by these miRNAs and decide on their functional relevance (Figure 6Aiii, Biii, Ciii). This functional analysis utilizing differentially expressed mRNA targets of miRNAs indicated that differentially expressed miRNAs may upregulate mRNAs linked to the Atherosclerosis signaling, Axon guidance, and Autophagy in POVPC vs. DMSO groups and downregulate the Activin inhibin signaling, AMPK signaling, and Endothelin-1 signaling (Figure 6Aiii). Also, in the siOCT4-DMSO vs. si-NT-DMSO groups, mRNA targets of miRNAs, which were found to be upregulated, included Axon guidance, DNA methylation and Transcriptional repression signaling. In contrast, downregulated mRNA targets included AMPK signaling, Adipogenesis pathway, and Acute myeloid leukemia signaling (Figure 6Biii). In the siOCT4-POVPC vs. siOCT4-DMSO groups, the upregulated miRNA targets included Cardiac hypertrophy signaling, Axon guidance signaling, and CREB signaling in neurons, whereas the downregulated miRNA targets included AMPK signaling, Actin cytoskeleton signaling, and 3-phosphoinositide biosynthesis (Figure 6Ciii).

#### Integrative analysis of differentially expressed lncRNA, miRNA, and mRNA in OCT4-deficient MASMC

To explore the ability of lncRNA to act as ceRNAs (to affect mRNA levels by sequestering common miRNAs that target both lncRNAs and mRNAs), we integrated upregulated lncRNAs/mRNAs with the downregulated miRNAs. For this analysis, we chose lncRNAs that were highly upregulated with a raw signal intensity of more than 100 (corresponding to the highly expressed genes in microarrays). Only lncRNAs listed in GENCODE and Ensemble or characterized by *Cabili et al.* (Yuan et al., 2022) were chosen for annotation purposes. We obtained data on miRNA binding to particular lncRNAs using LncBase v.2, included in DIANA Tools (Paraskevopoulou et al., 2016).

Three of the picked lncRNAs that were upregulated had more than one binding site for six downregulated miRNAs. Thirteen of the upregulated mRNAs were potential targets of these six miRNAs. Next, we connected these lncRNA/miRNAs pairs to the expression of mRNAs by functioning via potential ceRNA-mechanism (Table 1). In the POVPC vs. DMSO and siOCT4-POVPC vs. siOCT4-DMSO groups, ENSMUST00000140952 was shown to have seven and four potential binding sites for the downregulated mmu-miR-10a and mmu-miR-196a-1, correspondingly. Both miRNAs were previously linked to cell migration (Cui et al., 2021; Zeng and Li, 2014). These relationships may help to understand how ENSMUST00000140952 contributes to the upregulation of mRNAs such as *Hoxb6, Hoxb7, Hoxb4, Hoxb9, Hoxb5,* and *Hoxb3*, which are controlled by these miRNAs (Yang et al., 2018) (Table 1). In the siOCT4-DMSO vs. siNT-DMSO groups, the ENSMUST00000131663 lncRNA contained four binding sites for downregulated mmu-miR-486-5p and mmu-miR-96 recognized for controlling a number of the elevated mRNAs involved in proliferation such as *Aff3* (Lefevre et al., 2015)*, Rev1* (Chen et al., 2022), and *Eif5b* (Chukka et al., 2021), whereas the ENSMUST00000155531 lncRNA had four binding sites for downregulated mmu-miR-155 and mmu-miR-122 known to regulate mRNAs that were upregulated, such as *Zfp652, Gngt2, Phospho1,* and *Phb* (Table 1). Overall, these data indicate that upregulated lncRNAs may be able to sequester miRNAs in MASMCs, controlling the expression of their downstream target genes.

**TABLE 1:** List of Upregulated lncRNAs/mRNAs with potential downregulated miRNA targets.

LncRNA	Fold change	*p*-value	Micro RNA	No. of binding sites on lncRNA	Fold change	*p*-value
POVPC vs. DMSO						
ENSMUST00000140952	3.68	0.03	mmu-miR-10a	7	0.67	0.001
			mmu-miR-196a-1	4	0.01	0.001
siOCT4-DMSO vs. siNT-DMSO						
ENSMUST00000131663	3.03	0.001	mmu-miR-486-5p	4	0.89	0.014
			mmu-miR-96	4	0.53	0.001
ENSMUST00000155531	3.22	0.01	mmu-miR-155	4	0.79	0.016
			mmu-miR-122	4	0.46	0.001
siOCT4-POVPC vs. siOCT4-DMSO						
ENSMUST00000140952	2.59	0.04	mmu-miR-196a-1	4	0.01	0.001
			mmu-miR-10a	7	0.67	0.001
miRNA	mRNA					
mmu-miR-10a, mmu-miR-196a-1:	Hoxb6, Hoxb7, Hoxb 4, Hoxb9, Hoxb5, Hoxb3					
miR-486-5p, miR-96:	Aff3, Rev1, Eif5b					
mmu-miR-155, mmu-miR-122:	Zfp652, Gngt2, Phospho1, Phb					

Our analysis also found an inter-regulatory link between downregulated mRNA/lncRNA and upregulated miRNAs. In this instance, three of the selected downregulated lncRNAs, including ENSMUST00000151998, ENSMUST00000137236, and ENSMUST00000173605, had more than one binding site for nine of the upregulated miRNAs (Table 2). Eighty-eigh of the downregulated mRNAs were the targets of these nine miRNAs. In the POVPC vs. DMSO groups, ENSMUST00000151998 had 22 and 6 anticipated binding sites for the upregulated mmu-miR-15a-5p and mmu-miR-322-5p, respectively. These lncRNA/miRNA relationships provide a potential mechanism on how this lncRNA contributes to the downregulation of mRNAs that are regulated by these miRNAs (Table 2). The ENSMUST00000137236 lncRNA, in siOCT4-DMSO vs. siNT-DMSO, had 15 and 13 predicted binding sites for the upregulated mmu-miR-146a-5p/mmu-miR-146b-5p and mmu-miR-15b-5p, respectively. These relationships provide a potential mechanism on how this lncRNA contributes to the downregulation of mRNAs (Table 2). In the siOCT4-POVPC vs. siOCT4-DMSO groups, downregulated ENSMUST00000173605 had three predicted binding sites for mmu-miR-10a-5p/mmu-miR-10b-5p, and two predicted sites for mmu-miR-15a-5p/mmu-miR-15b-5p and mmu-miR-30a-5p (all upregulated miRNAs), which were reported to regulate cell migration and apoptosis (Shen et al., 2021; Li et al., 2022; Chen et al., 2020), and phagocytosis (Cho et al., 2019; Murinello et al., 2019) and were associated with downregulated mRNAs (Table 2).

**TABLE 2:** List of downregulated lncRNAs/mRNAs with potential upregulated miRNA targets.

LncRNA	Fold change	*p*-value	Micro RNA	No. of binding sites on lncRNA	Fold change	*p*-value
POVPC vs. DMSO						
ENSMUST00000151998	−2.88	0.01	mmu-miR-15a-5p	22	1.59	0.01
			mmu-miR-322-5p	6	2.01	0.01
siOCT4-DMSO vs. siNT-DMSO						
ENSMUST00000137236	−2.47	0.002	mmu-miR-146a-5p, mmu-miR-146b-5p	15	1.472	0.03
			mmu-miR-15b-5p	13	1.428	0.04
siOCT4-POVPC vs. siOCT4-DMSO						
ENSMUST00000173605	−3.80	0.026	mmu-miR-10a-5p, mmu-miR-10b-5p	3	1.79	0.003
			mmu-miR-15a-5p, mmu-miR-15b-5p	2	1.63	0.03
			mmu-miR-30a-5p	2	1.53	0.006
			mmu-miR-376a-3p	1	1.66	0.04
miRNA	mRNA					
mmu-miR-15a-5p:	Dedd, E2f3, Tmem178b, Capza2, Glud1, Sema3a, Cbfa2t3, Nr2c2, Ttc14, Clock					
mmu-miR-322-5p:	Rnf111, Rgs8, Slc4a4, E2f3, Arpp21, Cyb561a3, Htr4, Lcor, Akap7, Slc22a23, Acvr2b, Adrb2					
mmu-miR-146a-5p:	Traf6, Rsad2					
mmu-miR-146b-5p:	Kctd15, Zfp532, Strbp, Ehf, Gabrb1, Smad4, Angptl2, Sfpq, Primpol, Tlcd5, Btla, Dcp1a, Itm2b, Syt1, Traf6, Ar, Slamf1, Lipa, Znrf3, Card10					
mmu-miR-15b-5p:	Tspyl2, Gpr174, Tmem178b, Cyb561a3, Atxn1l, Clock, Ncl, Fgd4					
mmu-miR-10a-5p:	Mapre1, Epha5, Myt1l, Tiam1, Flt1, Nr6a1, Hoxa1, Prrx1, Xrn1, Mapkbp1, Hoxd10, Nr4a3, Rybp, Lpar2, Rhpn2, Hdac4					
mmu-miR-10b-5p:	Hoxd10, Iffo2, Usp45, Tiam1, Hoxb3, Tiam1					
mmu-miR-30a-5p:	Ccne2, Gramd2, Adam9, Itga6, Ppp3cb, Sema3a					
mmu-miR-376a-3p:	Bcl9l, Kmt2a, Zfp148, Cdc34, E2f3, Crygf, Snx10, Zdhhc23					

**FIGURE 7:**
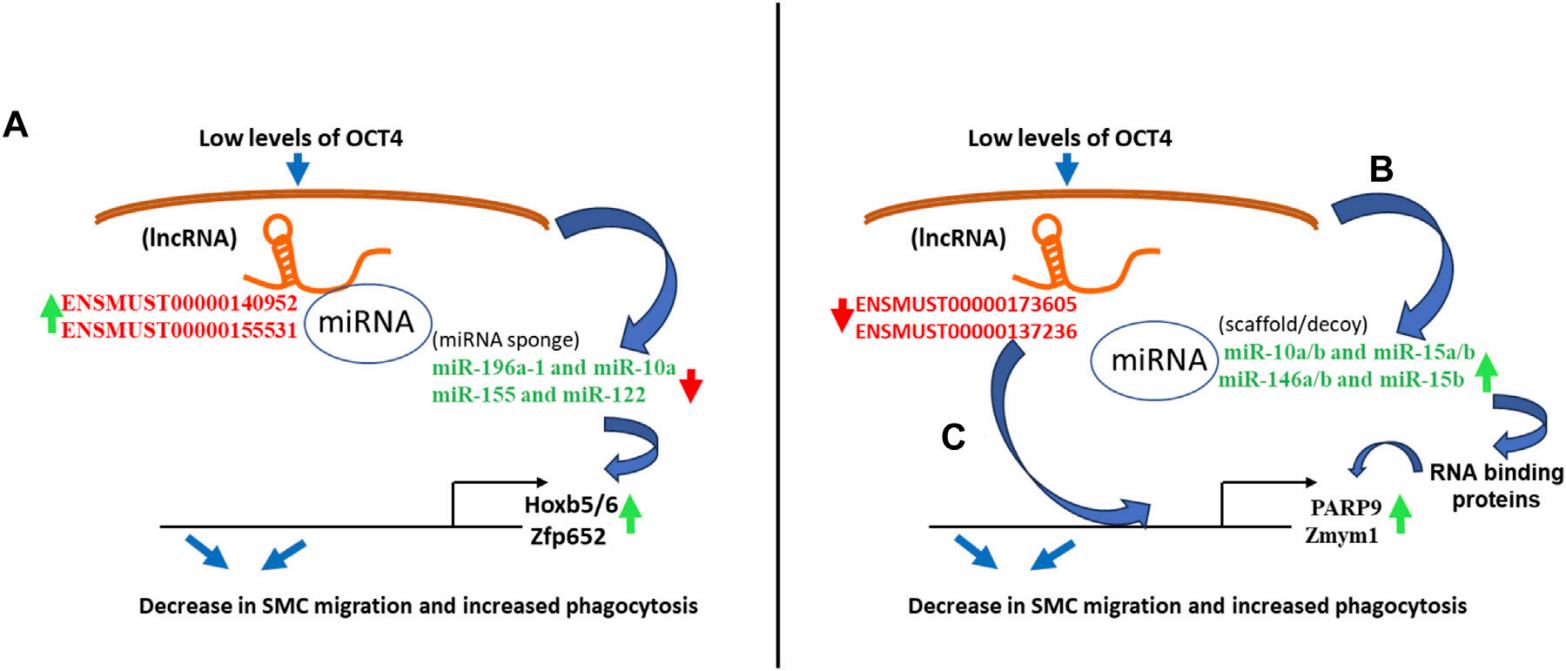
Schematic showing regulatory mechanisms obtained from the integrative analysis of the lncRNA-miRNA-mRNA interactions in OCT4 deficient SMCs. **(A)** Long non-coding RNAs (ENSMUST00000140952, ENSMUST00000155531) act as miRNA sponges (miR-196a-1 and miR-10a, miR-155 and miR-122) that regulate target gene expression (*Hoxb5/6*, Zfp652) **(B)** Decreases in lncRNAs (ENSMUST00000173605, ENSMUST00000137236) result in increased miRNA expression (miR-10a/b and miR-15a/b, miR-146a/b and miR-15b), which serve as scaffolding factors that “chaperone” RNA-binding proteins (RBPs) to form functional RNPs. In addition, or associated with this role, some small ncRNAs may act as molecular decoys, impairing the RBP-guided control of RNA fate by competing with other RNA substrates and, in turn, regulating the mRNA expression (*Parp9*, *Zmym1*) **(C)** Long non-coding RNAs (ENSMUST00000173605, ENSMUST00000137236) directly bind to mRNAs (*Parp9*, *Zmym1*) and regulate mRNA stability or competitively bind to mRNA to improve mRNA stability.

Our integrative analysis indicates that OCT4 is a critical regulator of lncRNAs and miRNAs and their cross-interactions in mouse aortic SMC. Together with our published and new functional studies about the role of OCT4 in SMC phenotypic transitions, we can conclude that OCT4-dependent lncRNA/miRNA/mRNA interactions might significantly contribute to SMC-dependent vascular pathologies, including atherosclerosis (Figure 7).

## Discussion

We previously demonstrated that the pluripotency factor OCT4 plays a critical regulatory role in vascular SMC phenotypic transitions *in vitro* and *in vivo*. Bulk RNAseq performed on *Oct4* knockout and wild-type MASMC revealed a robust dysregulation of gene expression in OCT4 deficient cells compared to control. However, nothing was known about the effects of OCT4 on non-coding RNAs or their potential contribution to OCT4-dependent responses in SMC. In this study, we performed an integrative bioinformatics analysis of the lncRNAs, miRNA, and mRNAs cross-interactions and predicted the potential role of these interactions in regulating OCT4-dependent phenotypic transitions of SMC. We utilized lncRNA/mRNA expression array and small RNA microarray to evaluate lncRNAs, miRNAs, and mRNA expression levels in mouse aortic SMC transfected with siRNA blocking *Oct4* (OCT4 deficient SMC). Our integrative analysis demonstrated an important role of OCT4 in regulating lncRNAs and miRNAs in vascular SMC and revealed several OCT4-dependent lncRNA/miRNA/mRNA associations in the differential regulation of pathways associated with cell migration, proliferation, and phagocytosis.

Our integrative analysis of the lncRNA/miRNA/mRNA interactions demonstrated another level of complexity in the molecular mechanisms responsible for vascular SMC phenotypic transitions. We previously showed that pluripotency factors OCT4 and Krϋppel-like factor 4 (KLF4) are the key transcriptional factors regulating SMC transitions *in vitro* and *in vivo*. Using SMC-lineage tracing and SMC-specific knockout mouse models, we found that OCT4 played an atheroprotective role in SMC, while KLF4 had the opposite atheropromoting role (Shankman et al., 2015; Cherepanova et al., 2016). Intriguingly, we found that KLF4 directly positively regulates OCT4 activation (Cherepanova et al., 2016), an observation that does not fit the opposite roles of these two transcriptional factors. Interestingly, previous studies in human embryonic stem cells showed that miR-145 is involved in a double-negative feedback loop along with OCT4 and KLF4 regulating balancing in cell pluripotency and differentiation (Xu et al., 2009). Our new data make it interesting to speculate that lncRNA/miRNA interactions might be involved in the OCT4/KLF4 crosstalk in SMC.

One of the top upregulated lncRNAs in MASMCs treated with the oxidized phospholipid POVPC and under OCT4 deficient (siOCT4-POVPC vs. siOCT4-DMSO) conditions was ENSMUST00000140952 localized in the nucleus, in the sense strand from the *Hoxb5/6* gene. HOXB5/6 has been associated with an increase in proliferation in breast cancer tissues and cell lines (Lee et al., 2015) and has been reported to promote the proliferation and migration of pancreatic cancer cells (Gao et al., 2020). Further studies are needed to determine the molecular relationship between *Hoxb5/6* and the adjacent lncRNA ENSMUST00000140952 in MASMC and atherosclerosis.

Long non-coding RNAs exhibit a faster evolutionary rate and are not as conserved across vertebrates as other RNA sequences. Among the limited number of lncRNAs conserved across species, their sequences are only partially preserved (Johnsson et al., 2014). While the field of non-coding RNA research is progressing towards revising potential models for lncRNA functional conservation, with a focus on short motif and structural domain analysis (Ross et al., 2021; Ross and Ulitsky, 2022), the notable degree of conservation observed in certain lncRNAs, such as the case of *Hoxb5os/HOXB3-A*S3 (Papaioannou et al., 2019; Degani et al., 2021), suggests their potential significance during development and physiological processes. Additionally, their dysregulation in various disease conditions may further underscore their functional relevance.

In addition, our analyses predicted associations between the gene/lncRNA pairs: *Zmym1*/ENSMUST00000137236 and *Parp9*/ENSMUST00000173605. The expression of the lncRNA, ENSMUST00000137236, was downregulated, and its associated mRNA, *Zmym1,* was upregulated in OCT4 deficient (siOCT4-DMSO vs. siNT-DMSO) MASMCs. The *Zmym1* gene has been reported to promote epithelial-to-mesenchymal transition and metastasis, cell growth and migration by inhibiting E-cadherin expression in gastric cancer (Yue et al., 2019). Downregulated ENSMUST00000173605 associated with upregulated *Parp9* (siOCT4-POVPC vs. siOCT4-DMSO). *Parp9* is reported to be involved in promoting the proliferation, survival, and chemotherapy resistance in lymphoma and prostate cancer, and its over-expression in human breast cancer is associated with cancer cell migration (Tang et al., 2018). In addition, PARP9 has been reported to regulate pro-inflammatory responses in macrophages (Iwata et al., 2016).

Analysis of differentially expressed miRNAs and their mRNA targets in OCT4 deficient cells revealed several upregulated miRNAs, including miR-10a/b, miR-15a/b, and miR-146a/b that target mRNAs involved in anti-migratory and pro-phagocytic signaling. Micro RNA-10a/b is implicated in the suppression of cardiac hypertrophy and cell survival (Stadthagen et al., 2013), while miR-15a/b has been implicated in other processes, such as suppression of cell survival and induction of apoptosis in chronic myeloid leukemia and endometrial cancer cells (Chen D. et al., 2017; Wang et al., 2017). Further, miR-146a is known to inhibit cancer migration, invasion, and metastasis by downregulating vascular endothelial growth factor (VEGF) through dual pathways in hepatocellular carcinoma cells (Sun et al., 2015; Yin et al., 2019).

It is known that lncRNAs control gene expression via sponging miRNAs, which in turn control mRNAs (Zhang et al., 2018). Several lncRNAs with binding sites for these downregulated anti-proliferative miRNAs were identified by our integrative bioinformatics analysis of the elevated lncRNA and mRNAs and the downregulated miRNAs in OCT4 deficient cells. We observed that ENSMUST00000140952 and ENSMUST00000155531 possessed binding sites for the miRNAs miR-196a-1 and miR-10a and miR-155 and miR-122, suggesting that they may be potential regulators of these miRNAs. Micro RNA-10a and miR-196a are reported to be oncogenic factors and are upregulated in various malignant tumor tissues (Bryant et al., 2012; Sun et al., 2012). Also, in non-small cell lung cancer (NSCLC) tissues, miR-196a was significantly upregulated, which in turn resulted in NSCLC cell migration and invasion, partially via downregulation of HOXA5 (Liu et al., 2012), and miR-155 has been implicated in with inflammation and cardiovascular diseases (Schroen and Heymans, 2012). In addition, previous reports characterized miR-155 and miR-122 as suppressors of cell migration and oxidative stress, including vascular SMC (Tong et al., 2023), whereas knockdown of miR-155-5p results in increased proliferation and migration of human brain micro-vessel ECs (Liu et al., 2015). Another significantly upregulated lncRNA, ENSMUST00000131663, had potential binding sites for miR-486-5p, which is known to function as a diagnostic marker for carotid artery stenosis and preventing endothelial dysfunction by inhibiting inflammation and oxidative stress (Zhu et al., 2022).

Similar to *Chinnappan et al.* (Chinnappan et al., 2019), who performed their integrative analysis on human pulmonary artery SMC, we found an association between the downregulated histone deacetylase 4 (*Hdac4*) and upregulated miR-10a/b (Table 2). This finding is very intriguing in that it suggests a critical role of this OCT4-dependent association in different types of SMC and adds another layer of complexity (chromatin modifiers) to the predicted OCT4-lncRNA-miRNA-regulatory network.

In summary, this study is the first attempt to elucidate novel potential OCT4-dependent interplay between lncRNAs, mRNAs, and miRNAs, which may play a role in phenotypic transitions of vascular SMC. We show that knockout of OCT4 results in robust dysregulation of lncRNA, miRNA, and their interactions, indicating that these interactions might be responsible for the observed OCT4-dependent functional effects (e.g., SMC migration, proliferation, and phagocytosis).

## Limitations of the study and future directions

However, our study has a few limitations. First, the lncRNA functions were characterized based on their primary sequence. However, recent reports suggest the additional role of tertiary structure in deciding lncRNA function (Mattick et al., 2023). Therefore, further functional and mechanistic studies are needed to investigate the role of the predicted lncRNA/miRNA/mRNA associations in SMC phenotypic transitions *in vitro* and *in vivo*. *Second*, although most of the non-coding RNAs were conserved in our study, it is possible that the OCT4-dependent molecular mechanisms associated with the murine phenotypic transition of vascular SMC may not be directly applicable to humans. Hence, additional experiments are required to validate the significance of the identified genes. *Third*, all experiments and analyses were performed *in vitro* using primary culture SMC. Therefore, further *in vivo* studies are needed to validate these results.

## Data Availability

The datasets presented in this study can be found in online repositories. Array data generated in this study were deposited into the Gene Expression Omnibus database [accession numbers GSE249982 (lnc/mRNA) and GSE249983 (small RNA/miRNA)]. We also used our previously published bulk RNAseq data (GSE75044).
