# Supplementary material for: Integrative analysis of the lncRNA-miRNA-mRNA interactions in smooth muscle cell phenotypic transitions
Source: Front Genet. 2024 Apr 10;15:1356558. doi: 10.3389/fgene.2024.1356558 (PMC11039880; doi:10.3389/fgene.2024.1356558)
Supplement: Supplementary file 3 [file DataSheet1.PDF]

Integrative analysis of the lncRNA-miRNA-mRNA Interactions in Smooth Muscle Cell Phenotypic Transitions

Aatish Mahajan[1], PhD; Junyoung Hong[1], PhD; Irene Krukovets[1], BS; Junchul Shin[1], PhD; Svyatoslav Tkachenko[2], PhD; Cristina Espinosa-Diez[3], PhD; Gary K. Owens[4], PhD; Olga A. Cherepanova[1], PhD*

[1]Department of Cardiovascular and Metabolic Sciences, Lerner Research Institute, Cleveland Clinic, Cleveland, OH, USA

[2]Department of Genetics and Genome Sciences, Case Western Reserve University, Cleveland, OH, USA

[3]Center for Molecular Medicine and Genetics, Wayne State University, Detroit, MI, USA

[4]Robert M. Berne Cardiovascular Research Center, University of Virginia, Charlottesville, VA, USA

Supplementary Figures

Supplementary Figure 1

A ENSMUST00000140952 (Hoxb5os)

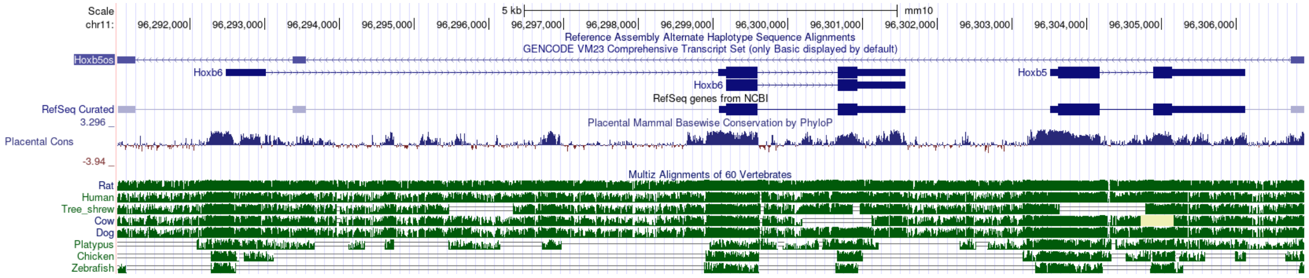

B ENSMUST00000155531 (Zfp652os)

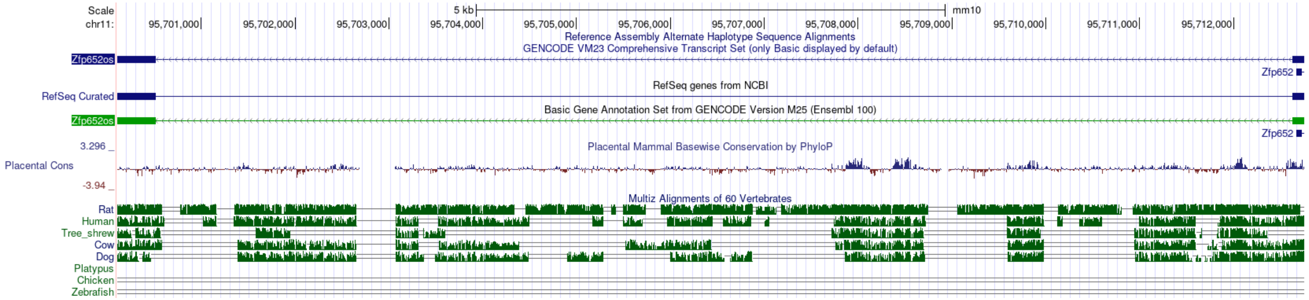

C ENSMUST00000173605 (Gm15564)

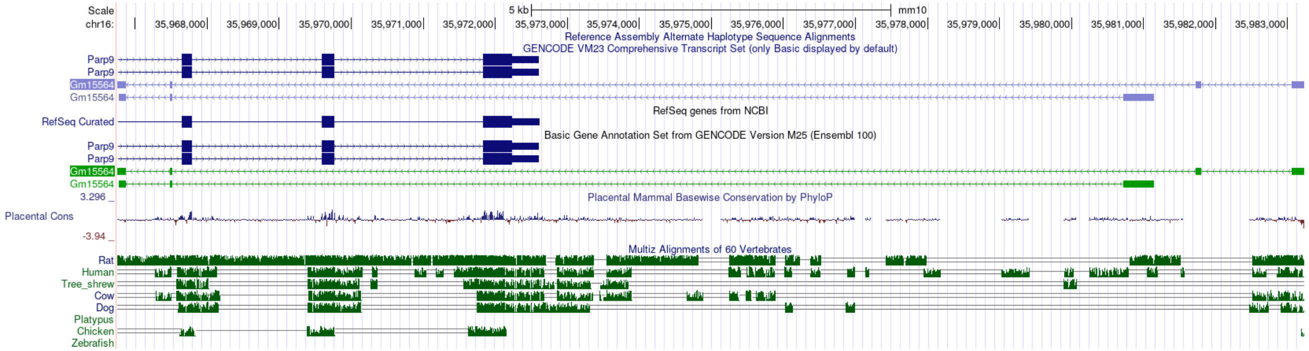

D ENSMUST00000137236 (Gm12940)

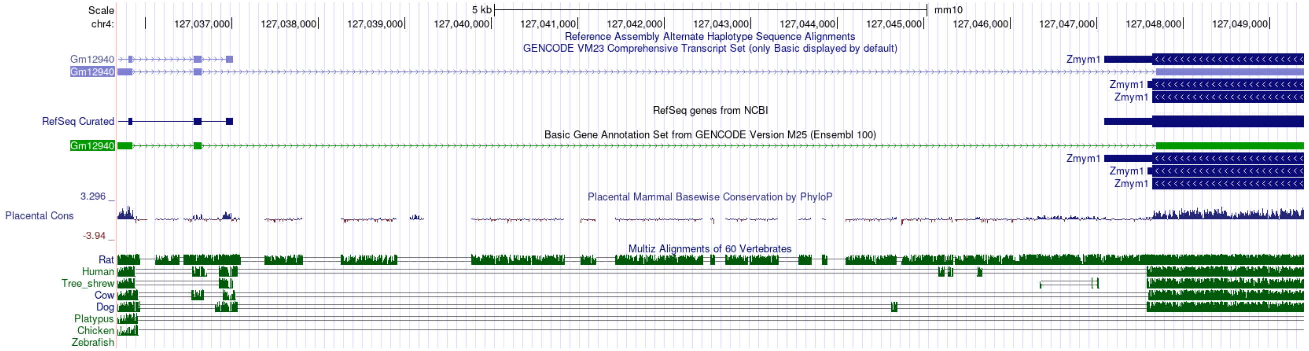

Supplementary Figure 1. Analysis of LncRNA sequence conservation in placental mammals and vertebrates. Representative images of UCSC browser alignment for (**A**) ENSMUST00000140952 (Hoxb5os), (**B**) ENSMUST00000155531 (Zfp652os), (**C**) ENSMUST00000173605 (Gm15564) and (**D**) ENSMUST00000137236 (Gm12940) genes. UCSC browser tracks show Reference assembly from GENCODE VM23 and NCBI. Conservation tracks show placental mammals and selected vertebrated evolutionary conservation analysis using phyloP method (PHAST (cshl.edu)). Conserved sites have positive scores (blue), while fast-evolving sites have negative scores (red).

Supplementary Figure 2

A

Description	Scientific Name	Max Score	Query Cover	E value	Per. Ident %	Accession
Homo sapiens HOXB cluster antisense RNA 3 (HOXB-AS3), transcript variant 4, long non-coding RNA	Homo sapiens	148	82%	2.00E-33	85.5	NR_033204.2
Homo sapiens homeobox B5 (HOXB5), mRNA	Homo sapiens	141	62%	9.00E-32	91.84	NM_002147.4
Homo sapiens homeobox C6 (HOXC6), transcript variant 2, mRNA	Homo sapiens	132	63%	2.00E-28	89	NM_153693.5
Homo sapiens homeobox C6 (HOXC6), transcript variant 1, mRNA	Homo sapiens	132	63%	2.00E-28	89	NM_004503.4
Homo sapiens homeobox A6 (HOXA6), mRNA	Homo sapiens	123	62%	3.00E-26	87.76	NM_024014.4
Homo sapiens homeobox A7 (HOXA7), mRNA	Homo sapiens	123	63%	9.00E-26	87	NM_006896.4
Homo sapiens homeobox B4 (HOXB4), mRNA	Homo sapiens	123	63%	9.00E-26	87	NM_024015.5
Homo sapiens HOXA cluster antisense RNA 3 (HOXA-AS3), transcript variant 2, long non-coding RNA	Homo sapiens	123	63%	9.00E-26	87	NR_038832.1
Homo sapiens HOXA cluster antisense RNA 3 (HOXA-AS3), transcript variant 1, long non-coding RNA	Homo sapiens	123	63%	9.00E-26	87	NR_038831.1
Homo sapiens HOXB cluster antisense RNA 3 (HOXB-AS3), transcript variant 6, long non-coding RNA	Homo sapiens	104	47%	2.00E-20	90.67	NR_110329.1
Homo sapiens HOXB cluster antisense RNA 3 (HOXB-AS3), transcript variant 3, long non-coding RNA	Homo sapiens	101	46%	3.00E-19	90.41	NR_033203.1

B

```
Hoxb5os-201 cDNA     1    CTTTCTTCCACTTCATGCGACGGTTCTGGAACCAGATTTT  40
                          |||| |||||||||||||| |||||||||||||| || ||
HOXB-AS3-204 cDNA    41   CTTT-TTCCACTTCATGCGTCGGTTCTGGAACCATATCTT  79

Hoxb5os-201 cDNA     41   GATCTGACGCTCGGACAGGCAAAGCGCGTGGGCGATCTCG  80
                          |||||| | ||| | |||||| || ||||| |||||||||
HOXB-AS3-204 cDNA    80   GATCTGCCTCTCCGTCAGGCACAGGGCGTGCGCGATCTCG  119

Hoxb5os-201 cDNA     81   ATACGTCGCCGCCGGGTCAGACGCCTCTTCGCCCTAGGCT  120
                          || || |||||||| ||||| ||  || || ||||  ||
HOXB-AS3-204 cDNA    120  ATGCGCCGCCGCCGCGTCAGCCGTTTCCTCTCCCT-CGCC  158

Hoxb5os-201 cDNA     121  GGCCTCTGCGGAGATTCCAGGCCCCACAGAGACCAGGA  158
                          |||||| ||||||||||||||||| | ||| |||||||
HOXB-AS3-204 cDNA    159  GGCCTCGGCGGAGATTCCAGGCCCTATAGAAACCAGGA  196
```

C Gene : ENSMUSG00000085645 (Hoxb5os)

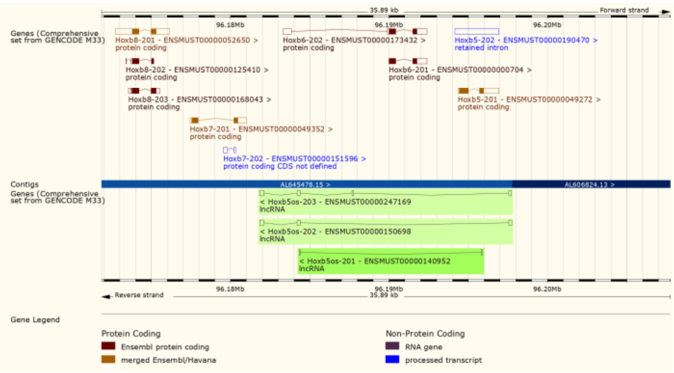

D Gene : ENSG00000233101 (HOXB-AS3)

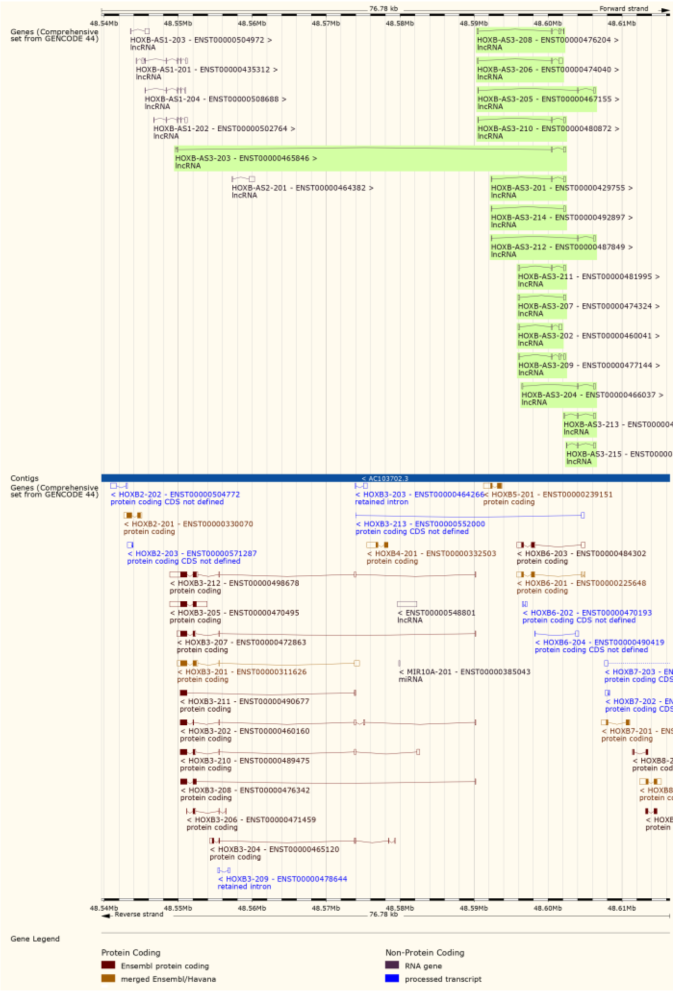

Supplementary Figure 2. Characterization of human ortholog for ENSMUST00000140952. (**A**) Summary table of top score Blastn alignments for ENSMUST00000140952 against the human genomic and transcript database. (**B**) Representative alignment of ENSMUST00000140952 (Hoxb5os-201) against the potential human ortholog transcript HOXB3-AS3-204. (**C**) Genomic location of ENSMUST00000140952 associated gene Hoxb5os. (**D**) Genomic location of the human potential human ortholog gene ENSG00000233101 (HOXB-AS3).

Supplementary Figure 3

A

Description	Scientific Name	Max Score	Query Cover	E value	Per. Ident %	Accession
Homo sapiens ZNF652 antisense RNA 1 (ZNF652-AS1), transcript variant 1, long non-coding RNA	Homo sapiens	157	19%	2.00E-35	84.25	NR_110882.1
PREDICTED: Homo sapiens zinc finger protein 652 (ZNF652), transcript variant X8, mRNA	Homo sapiens	75.2	8%	6.00E-11	86.89	XM_047435629.1
Homo sapiens zinc finger protein 652 (ZNF652), transcript variant 2, mRNA	Homo sapiens	75.2	8%	6.00E-11	86.89	NM_014897.2

B

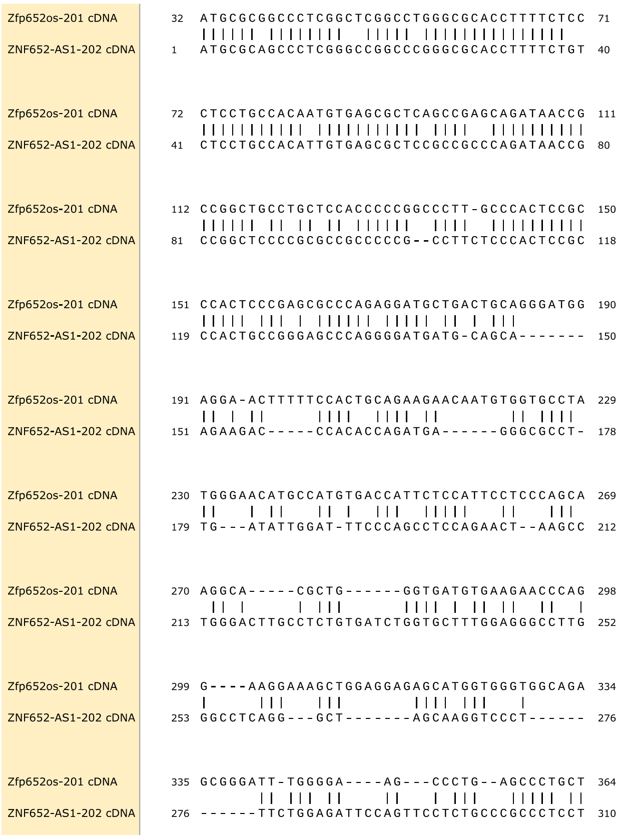

C

Gene : ENSMUSG00000086191 (Zfp652os)

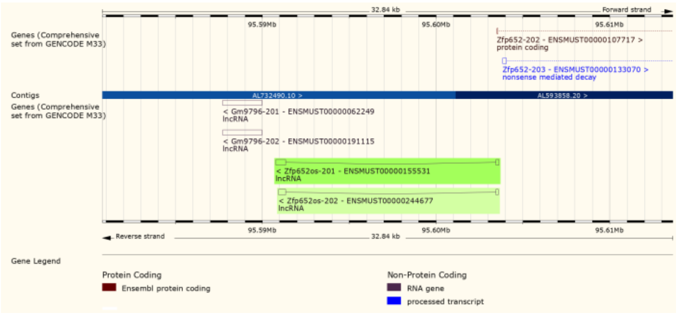

D

Gene : ENSG00000198740 (ZNF652)

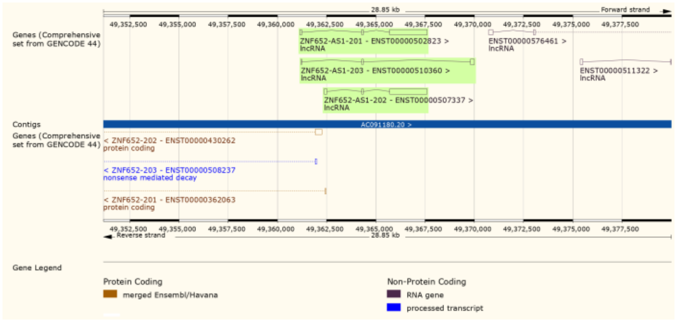

Supplementary Figure 3. Characterization of human ortholog for ENSMUST00000155531. (**A**) Summary table of top score Blastn alignments for ENSMUST00000155531 against the human genomic and transcript database. (**B**) Representative alignment of ENSMUST00000155531 (Zfp652os-201) against the potential human ortholog transcript ZNF652-AS1-202. (**C**) Genomic location of ENSMUST00000155531 associated gene Zfp652os. (**D**) Genomic location of the human potential human ortholog gene ENSG00000248714 (ZNF652-AS1).

Supplementary Figure 4

A

Description	Scientific Name	Max Score	Query Cover	E value	Per. Ident %	Accession
Homo sapiens zinc finger MYM-type containing 1 (ZMYM1), transcript variant 1, mRNA	Homo sapiens	1349	86%	0	77.65	NM_001289088.2
Homo sapiens zinc finger MYM-type containing 1 (ZMYM1), transcript variant 6, mRNA	Homo sapiens	1349	86%	0	77.65	NM_001319955.2
Homo sapiens zinc finger MYM-type containing 1 (ZMYM1), transcript variant 3, mRNA	Homo sapiens	1349	86%	0	77.65	NM_001289089.2
Homo sapiens zinc finger MYM-type containing 1 (ZMYM1), transcript variant 2, mRNA	Homo sapiens	1349	86%	0	77.65	NM_001289091.2
Homo sapiens zinc finger MYM-type containing 1 (ZMYM1), transcript variant 4, mRNA	Homo sapiens	1349	86%	0	77.65	NM_001289090.2
Homo sapiens zinc finger MYM-type containing 1 (ZMYM1), transcript variant 5, mRNA	Homo sapiens	1349	86%	0	77.65	NM_024772.5

B

```
Gm12940-202 cDNA   327  GTTTCTCTTGTCCCATGGTACGAAGTAAGTATGTTTTAAG  366
                        ||||| |||           |||||     ||  ||| ||
ZMYM1-206 cDNA     5    GTTTCGCTT-----------CGAAG-----ATTGTTTCAG  28

Gm12940-202 cDNA   367  ATGAGACAGCGT-----GGAAAATGACTTCTCATTATTTG  401
                        |      |||||     ||     | | ||
ZMYM1-206 cDNA     29   A------AGCGTTGGGCGG-----GGCGTC---------C  48

Gm12940-202 cDNA   402  CTGA-AGTAATTGGCCAAGATAAAGCAATAT-GCAACAG-  438
                        |||| || |||| |            ||| | | ||| |
ZMYM1-206 cDNA     49   CTGAGAGAAATTAG------------AATCTGGAAACTGT  76

Gm12940-202 cDNA   438  -CTTCGTGATACAAGGAA--TATTATTGTGGA----GAGC  471
                         ||||  |    ||| ||   |||| | ||||    |||
ZMYM1-206 cDNA     77   TCTTCAGG----AAGAAACCCATTAGTTTGGAACTGGAGA  112

Gm12940-202 cDNA   472  ATGC---TGAATAAACAAAGAGCCTAGGTCAATGAAGCTT  508
                        || |   || ||      |||  |||    |||||
ZMYM1-206 cDNA     113  ATTCCTTTGCAT-----CAGATACTA---AAATGA-----  139

Gm12940-202 cDNA   509  AAGGACCCATCCTCTGCGATGAAG--TTGAAACCTGCATA  546
                        ||| |||    || |  | || ||  |||       ||||
ZMYM1-206 cDNA     140  AAGAACC---ACTTTTAGGTGGAGTATTG------TCATA  170

Gm12940-202 cDNA   547  CTGCCGGTAAAA--TCGGAGCTC--GGGAATGATGTCTG-  581
                             || || |   | || |||  | ||||||   |||
ZMYM1-206 cDNA     170  -----GGCAACAGTCCAGAACTCAGGAGAATGA--ACTGA  203

Gm12940-202 cDNA   581  -CATCAA-GCT-TATAGAATTCCTG-GATGTGCTTGGCTG  617
                          || || ||| | |    || | | || ||||||  | |
ZMYM1-206 cDNA     204  AAATAAATGCTGTGT----TTTCAGAGA-GTGCTTCACAG  238

Gm12940-202 cDNA   618  TTG-CTTCATTTAATGGCTCATTCCACTT----AAGTAAC  652
                        ||| || ||      ||  |||||  |||      | | |
ZMYM1-206 cDNA     239  TTGACTGCA------GG--CATTCAGCTTTCTCTGGCATC  270
```

C

Gene : ENSMUSG00000085334 (Gm12940)

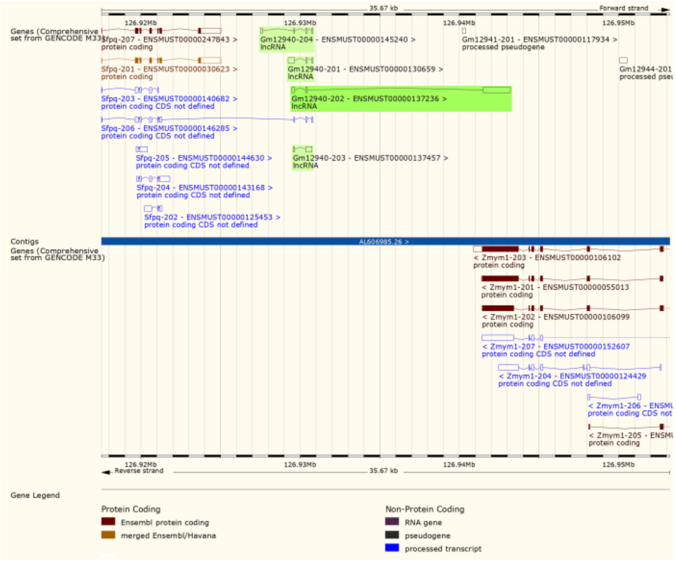

D

Gene : ENSG00000197056 (ZMYM1)

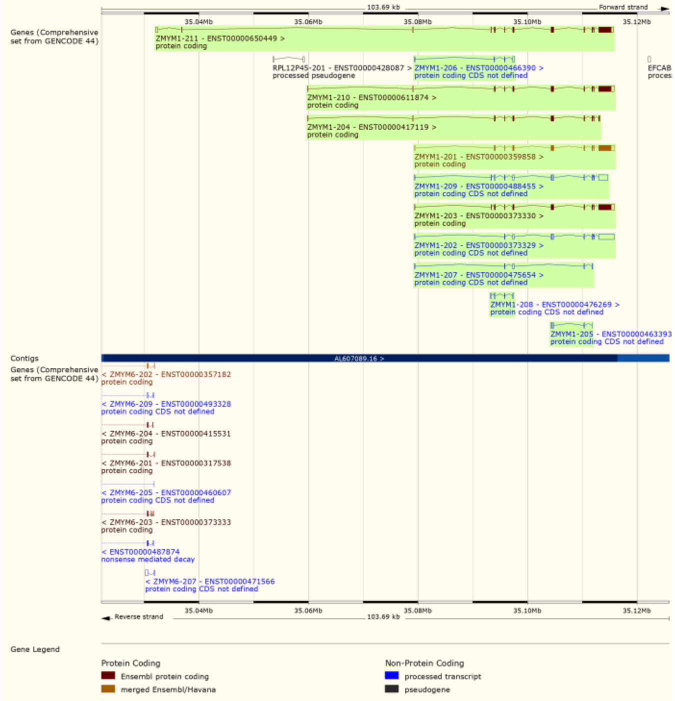

Supplementary Figure 4. Characterization of human ortholog for ENSMUST00000137236. (**A**) Summary table of top score Blastn alignments for ENSMUST00000137236 against the human genomic and transcript database. (**B**) Representative alignment of ENSMUST00000137236 (Gm12940-202) against the potential human ortholog transcript ZMYM1-206. (**C**) Genomic location of ENSMUST00000137236 associated gene Gm12940. (**D**) Genomic location of the human potential human ortholog gene ENSG00000197056 (ZMYM1). Protein coding transcripts are represented in red while processed transcripts with non-defined coding sequence (CDS) are represented in blue.

Supplementary Figure 5

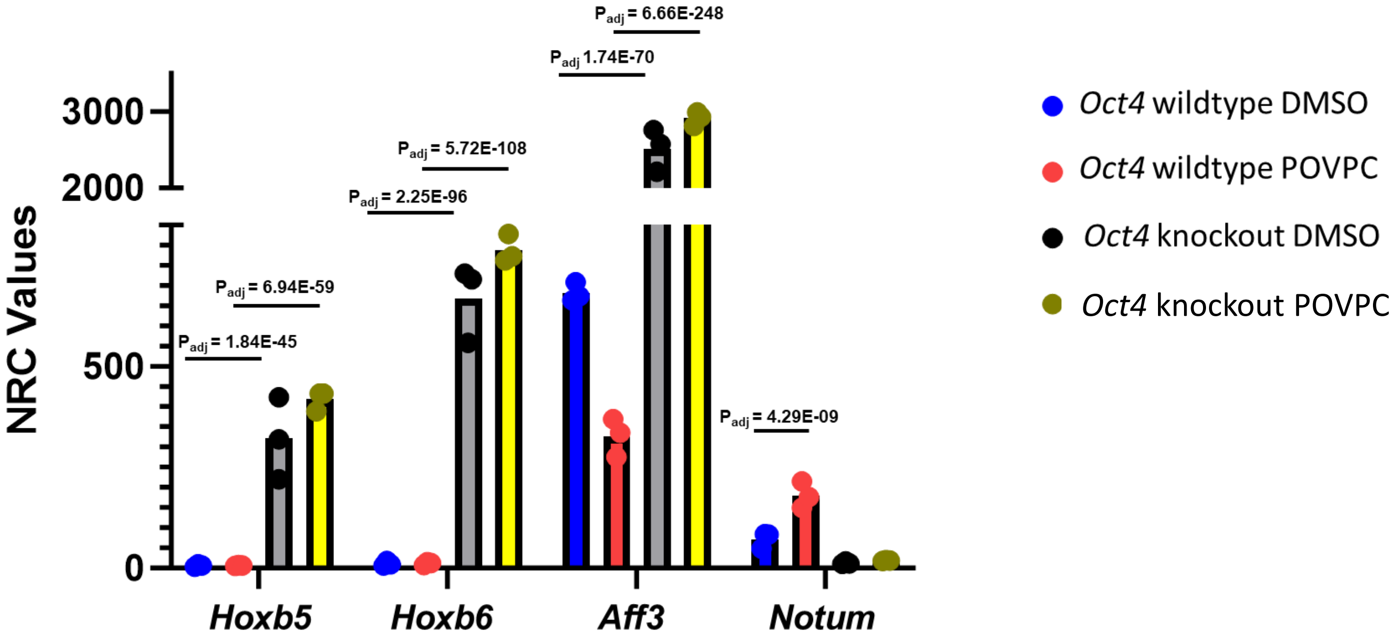

Supplementary Figure 5. Normalized read count (NRC) values for *Hoxb5*, *Hoxb6*, *Aff3*, and *Notum* from previously published bulk RNAseq on *Oct4* wildtype and knockout mouse aortic SMC treated with POVPC (GSE75044), *n* = 3. P adj = P adjusted.

Supplementary Figure 6

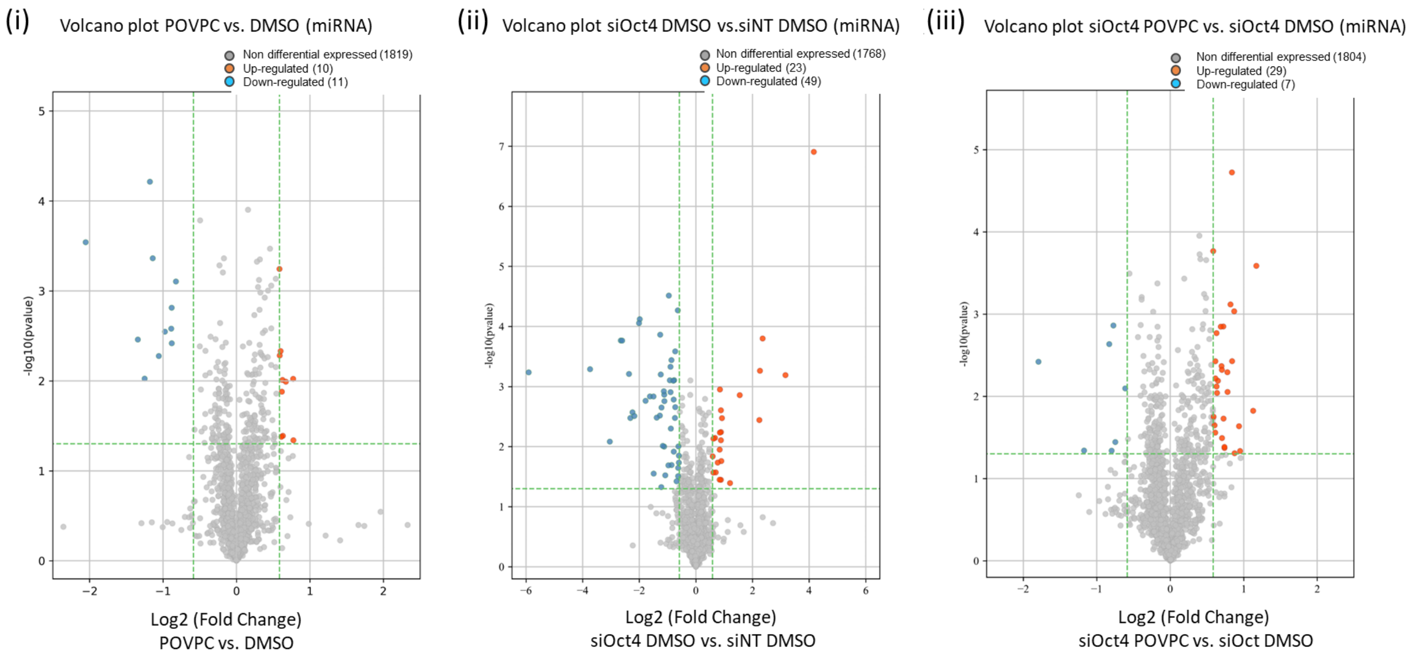

Supplementary Figure 6. Volcano plot of significantly dysregulated miRNAs for POVPC vs. DMSO treated SMCs (**i**), siOCT4-DMSO vs. siNT-DMSO (**ii**) and siOCT4-POVPC vs. siOCT4-DMSO (**iii**).
